# Catalytic efficiency of Cu-MOFs: HKUST-1 and CuBDC for the protodeboronation of aryl boronic acids

**DOI:** 10.1039/d5ra04172d

**Published:** 2025-08-21

**Authors:** Yudha P. Budiman, Muhamad R. S. Sidik, Muhamad Diki Permana, Kansy Haikal, Iis I. Widiyowati, Yessi Permana, Ubed S. F. Arrozi, Wirawan Ciptonugroho, Tri Mayanti, Allyn P. Sulaeman, Juliandri, Witri Wahyu Lestari

**Affiliations:** a Department of Chemistry, Faculty of Mathematics and Natural Sciences, Universitas Padjadjaran 45363 Sumedang Indonesia y.p.budiman@unpad.ac.id; b Special Educational Program for Green Energy Conversion Science and Technology, Integrated Graduate School of Medicine, Engineering, and Agricultural Sciences, University of Yamanashi Kofu 400-8511 Japan; c Center for Crystal Science and Technology, University of Yamanashi Kofu 400-8511 Japan; d Inorganic and Physical Chemistry Research Division, Faculty of Mathematics and Natural Sciences, Institut Teknologi Bandung Bandung 40132 Indonesia; e Department of Chemistry, Faculty of Mathematics and Natural Sciences, State University of Malang 65145 Malang Indonesia; f Department of Chemistry, Faculty of Mathematics and Natural Sciences, Universitas Sebelas Maret Surakarta 57126 Indonesia

## Abstract

This study investigates the catalytic potential of copper-based metal–organic frameworks (Cu-MOFs), specifically HKUST-1 and CuBDC, for the protodeboronation of aryl boronic acids. Protodeboronation, was explored under various bases, atmospheres, and substrates. Optimal conditions using K_2_CO_3_ as the base and an oxygen atmosphere yielded up to 98% product with HKUST-1. While CuBDC also exhibited catalytic activity, its yields were slightly lower under identical conditions. Substrate size and substituent effects played a crucial role, with bulkier substrates favoring higher yields. Recyclability tests confirmed that both Cu-MOFs retained catalytic activity over three cycles, despite some structural changes. These findings demonstrate Cu-MOFs as promising heterogeneous catalysts for controlled protodeboronation.

## Abstract 2

Cu-MOFs (HKUST-1 and CuBDC) effectively catalyzed the protodeboronation of arylboronic acids, giving up to 98% and 95% yields. Both catalysts remained active for three recycling cycles.

## Graphical abstract

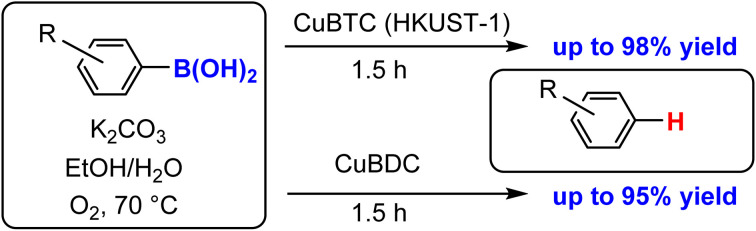

## 1 Introduction

**Scheme 1:**
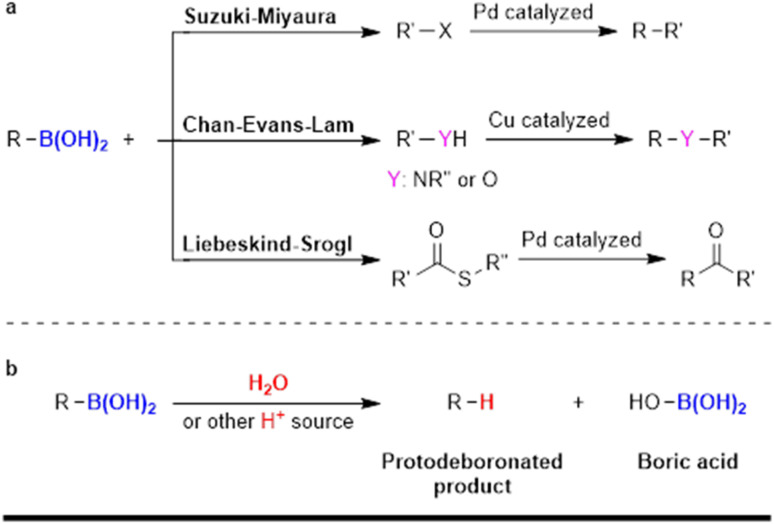
(a) Boronic acids utilized in coupling reactions and (b) protodeboronation of boronic acids.

Organoboron compounds are pivotal in organic synthesis due to their unique reactivity, high stability, and low toxicity,^1,2^ making them easy to handle without requiring special precautions.^3^ Their versatility spans numerous applications, including their use as nucleophilic substrates in coupling reactions,^4,5^ polymer precursors,^6^ Lewis acid catalysts,^7,8^ chemosensors,^9^ fluorescent probes,^10^ antibacterial agents,^11^ and functional materials.^12^ Boronic acid substrates and their derivatives have garnered significant attention, particularly in transition metal-catalyzed cross-coupling reactions renowned for forming strong carbon–carbon or carbon–heteroatom bonds, such as the Suzuki–Miyaura,^13^ Chan–Evans–Lam,^14–16^ and Liebeskind–Srogl reactions (Scheme 1a).^17^

Despite their utility, these reactions often encounter a competing pathway known as protodeboronation, where the carbon–boron bond is replaced with a carbon–hydrogen bond (Scheme 1b).^18^ This side reaction is typically undesirable, as it reduces the yield of the desired coupling product, thereby impacting the overall efficiency of the reaction.^19^

The first detailed study of this process dates back to 1930, when Ainley and Challenger observed that phenylboronic acid in water at 140–150 °C formed benzene after 40 h in the presence of stoichiometric amounts of salts (ZnCl_2_, CrBr_3_, CuSO_4_).^20^ Its synthetic potential was later explored by Brown and Zweifel in 1961.^21^ Since then, protodeboronation has evolved from being viewed as a side reaction to becoming an intentional step in certain synthetic pathways. For instance, Lai *et al.* specifically studied a palladium-catalyzed protodeboronation of arylboronic acids under basic conditions and a nitrogen atmosphere.^22^ In 2011, Elford *et al.* utilized the protodeboronation of boronic acid esters as a pivotal step in the synthesis of the natural product (+)-erogorgiaene.^23^ In the same year, Veguillas *et al.* demonstrated the synthesis of quinonyl boronic acid derivatives, where the boronic acid group played a critical role in initiating Friedel–Crafts alkylation *via* protodeboronation.^24^ Further developments were reported in 2013 by Lee *et al.*, who synthesized *o*- and *m*-phenols using phenylboronic acid as a precursor, with the boronic acid group serving as a temporary blocking or directing group before being removed through protodeboronation.^25^ In 2014, Lozada *et al.* studied a variety of electron-deficient and heteroarylboronic acids subjected to protodeboronation under basic conditions.^26^ More recently, Budiman *et al.* reviewed the influence of *o*-fluoro substituents on the reactivity of arylboronic acids in basic aqueous conditions, demonstrating how functional groups can enhance the likelihood of protodeboronation.^27^ These milestones underscore the growing importance of understanding and controlling protodeboronation, not only to minimize its occurrence as an undesirable side reaction but also to harness its potential as a deliberate tool in organic synthesis.

**Scheme 2:**
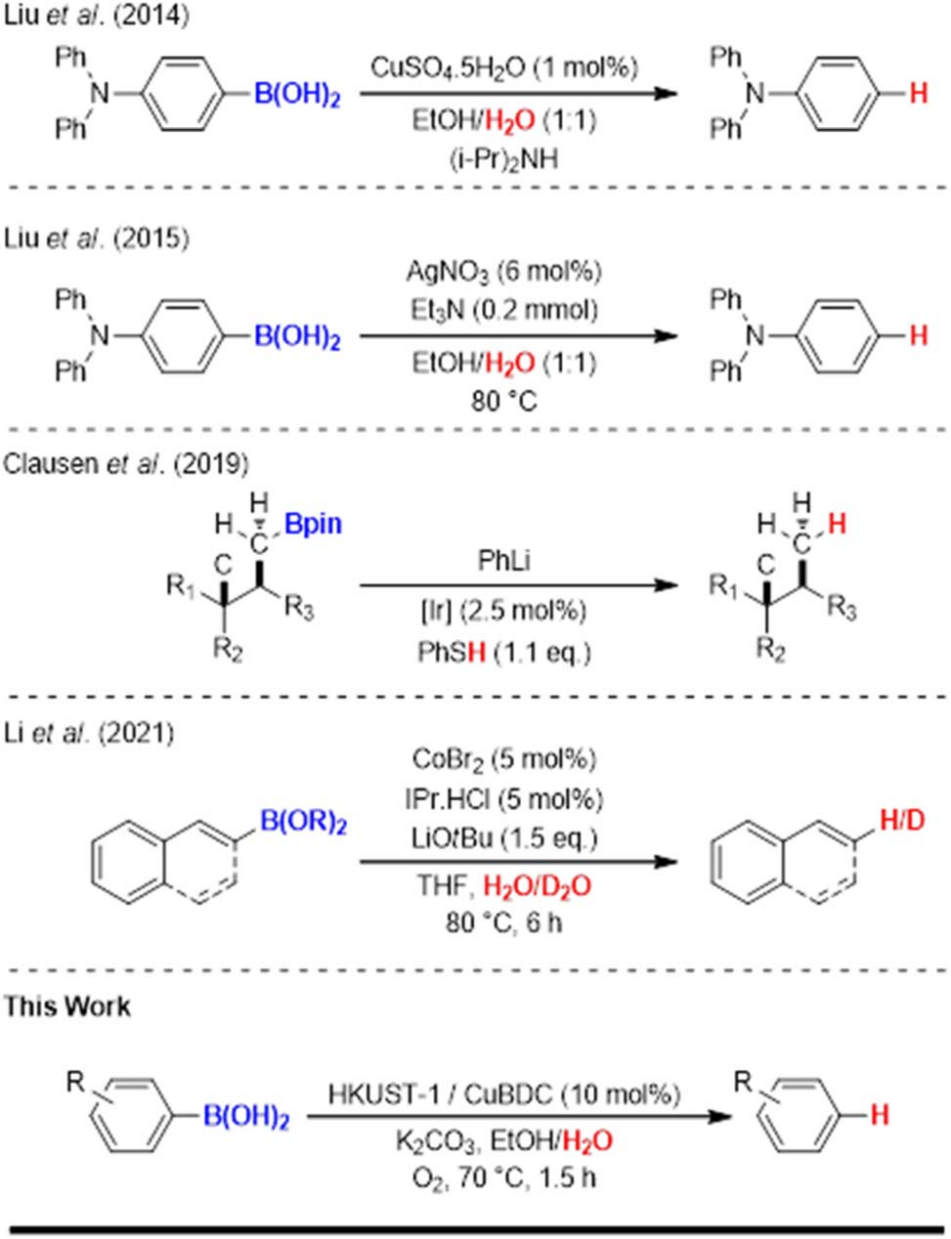
Transition metals catalyzed protodeboronation reactions.

To the best of our knowledge, the use of metal catalysts for protodeboronation has been extensively explored with metal salt systems such as copper,^28^ silver,^29^ iridium,^30^ and cobalt,^31^ among others (Scheme 2). In contrast, metal–organic frameworks (MOFs) present an emerging class of recyclable heterogeneous catalysts with significant potential, yet their application in protodeboronation reactions remains unexplored. The combination of hybrid inorganic–organic building blocks forming coordinated porous frameworks and the intrinsic rigidity of these frameworks imparts a wide range of functionalities with desirable properties.^32^ These features include active metal centers, adjustable pore sizes with high porosity, large surface areas, building block versatility, and flexible topological designs.^33^ MOFs have attracted considerable attention in organic synthesis due to their role as versatile heterogeneous catalysts with capabilities in catalyzing reactions such as Friedel–Crafts alkylation,^34,35^ Diels–Alder,^36^ cyclization,^37^ Michael addition,^38^ Claisen–Schmidt condensation,^39^ and CO_2_ cycloaddition.^40^ The adaptable structures of MOFs, both pre- and post-synthesis, enable precise control over their secondary building units (SBUs), pore functionalization, and the abundance of active sites resulting from higher metal content.^41^ These features have the potential to tailor the type of MOF catalyst needed for certain reactions. It is also important to underline that due to the inherent complexity of MOF structures, fully understanding the mechanisms of MOF catalytic systems is a challenging task, as both the metal nodes and organic linkers, as well as pore interactions, could play a role in providing catalytic sites.^42^

Building on our previous work optimizing HKUST-1 as a catalyst for the homocoupling of arylboronic acids,^43^ we now report the protodeboronation of arylboronic acids catalyzed by copper-based MOFs. Specifically, we employed HKUST-1 and CuBDC as catalysts. Both MOFs feature copper as the central metal site; however, they differ in their organic linkers. HKUST-1 utilizes benzenetricarboxylic acid (H_3_BTC),^44^ forming a three-dimensional framework, while CuBDC incorporates benzenedicarboxylic acid (H_3_BDC),^45^ resulting in a two-dimensional structure. This study explores the catalytic potential of these frameworks in protodeboronation reactions of arylboronic acids.

## 2 Experimental

### 2.1 Synthesis of HKUST-1

The synthesis of HKUST-1 was performed with a slightly modified electrochemical cell and solvent composition according to the literature procedure,^46^ which was optimized by our previous research.^47^ 1.06 g (5 mmol) of 1,3,5-H_3_BTC and 0.33 g (1 mmol) of tetra-butylammonium *tetra*-fluoroborate (TBATFB) were dissolved in 50 mL of solvent mixture water : ethanol (1 : 1) and stirred for 15 min prior to electrolysis. After the formation of a homogenous solution, two copper electrodes (with the same area, *i.e*. 10.5 cm^2^) were placed into the electrochemical cell. The system was set up with regulated DC power supply ATTEN TPR 3005T-3C under a constant voltage (15 V) at room temperature and kept for 1.5 h for complete reaction. The obtained sky-blue precipitate of HKUST-1 [Cu_3_(BTC)_2_·3H_2_O] (0.801 g – 53%, based on ligand) was then collected by filtration and washed three times with ethanol and dried at room temperature. The compounds were then activated at 120 °C prior to the experiment. Infrared spectra (KBr pellet, *ν*/cm^−1^, electro-synthesized HKUST-1): 3489–3386 (br), 1619 (vs), 1567 (s), 1447 (s), 1373 (vs), 1246 (s), 1187 (m), 1112 (m), 730 (vs), 491 (w).

### 2.2 Synthesis of CuBDC

The synthesis of CuBDC followed a procedure from literature.^45^ An equimolar quantity of 1.053 g copper nitrate trihydrate (Cu(NO_3_)_2_·3H_2_O) and 0.724 g terephthalic acid (C_6_H_4_(CO_2_H)_2_) were dissolved in 87 mL of dimethylformamide (DMF). This solution was placed in a closed scintillation flask in an oven at 110 °C for 36 h. Small blue precipitated crystals were visible inside the flask upon removal from the oven. After repeated centrifugation and washing, the light blue crystals of CuBDC was obtained. The compounds were then activated at 120 °C prior to the experiment.

### 2.3 Procedure of protodeboronation of aryl boronic acids

The reaction was carried out in a Schlenk tube by adding 10 mol% of the HKUST-1 or CuBDC catalyst, followed by 0.4 mmol of the arylboronic acid reactant and 1 equivalent of base. The Schlenk tube was then sealed with a septum, evacuated, and heated with a heat gun to re-activate the catalyst. Subsequently, a mixture of ethanol and water (1 : 1) was added *via* syringe modified with a cannula.

For reactions under controlled atmospheres, N_2_ or O_2_ was introduced by inflating a balloon attached to the syringe with the respective gas, while reactions in open air were performed by removing the septum from the Schlenk tube. The reaction was carried out at 70 °C for 1.5 h.

The reaction products were extracted using brine water (saturated NaCl solution, 2 mL) and ethyl acetate (2 mL), and washed with ethyl acetate up to three times. The ethyl acetate layer was accumulated, dried over anhydrous Na_2_SO_4_, filtered, and purified *via* column chromatography using *n*-hexane as the eluent to isolate the protodeboronation product. The extracted ethyl acetate layer containing the protodeboronation products were directly transferred to a vial after adding an equimolar amount of mesitylene (0.4 mmol) before being analyzed by GC-MS (Agilent 7890A) for its GC yield. Mesitylene was used as an internal standard to compare the GC response of the obtained products.

For catalyst recycling procedure, after extraction, the aqueous layer was centrifuged up to three times. The water was then decanted to separate it from the precipitated catalyst, which was recovered for reuse in another run.

## 3 Results and discussion

### 3.1 Effect of base

It is well-established that bases play a crucial role in enhancing the reactivity of protodeboronation,^22,27,48^ which is why we began by examining the effect of different bases on the model protodeboronation reaction of 4-*tert*-butylphenylboronic acid using either HKUST-1 or CuBDC (Table 1). As a control for the model reaction, we first tested the conditions without the Cu-MOF catalyst or a base, which produced no detectable amount of the protodeboronation product (Table 1, entry 1). Introducing 1 equivalent of K_2_CO_3_ without a catalyst improved the yield to 31% (Table 1, entry 2). In contrast, when the catalysts were used without a base, the reaction yielded only 12% of the protodeboronation product with HKUST-1 and 19% with CuBDC (Table 1, entries 3 and 12). These results underscore the crucial role of a base in promoting the protodeboronation reaction. The arylboronic acid is converted into an arylboronate anion [ArB(OH)_3_]^−^, which which facilitates protodeboronation.^28^

**Table 1:** Screening of different bases on the protodeboronation reaction of 4-*tert*-butylphenylboronic acid^a^

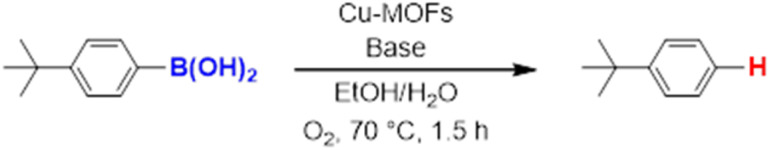				
Entry	Catalyst	Base	Base equivalent	Yield (%)
1	—	—	—	—
2	—	K_2_CO_3_	1.0	31
3	HKUST-1	—		12
4		KHCO_3_	1.0	29
5		CH_3_COOK	1.0	36
6		Na_2_CO_3_	1.0	54
7		NEt_3_	1.0	78
8		CsF	1.0	65
9		K_2_CO_3_	1.0	98
10		K_2_CO_3_	0.5	69
11		K_2_CO_3_	0.2	46
12	CuBDC	—	—	19
13		KHCO_3_	1.0	38
14		CH_3_COOK	1.0	53
15		Na_2_CO_3_	1.0	47
16		NEt_3_	1.0	83
17		CsF	1.0	51
18		K_2_CO_3_	1.0	95
19		K_2_CO_3_	0.5	52
20		K_2_CO_3_	0.2	47

a Reaction conditions: 4-*tert*-butylphenylboronic acid (0.4 mmol), Cu-MOFs (10 mol%), EtOH/H_2_O (1 mL/1 mL), O_2_, 70 °C, 1.5 h. GC yields were reported with mesitylene as an internal standard.

To further investigate the impact of the different Cu-MOF catalysts used in the model, a series of bases were tested using both Cu-MOF catalysts. The choice of base had significant impact on the outcome, as the HKUST-1-catalyzed reactions with bases such as KHCO_3_, CH_3_COOK, Na_2_CO_3_, NEt_3_, and K_2_CO_3_ produced low to excellent yields (Table 1, entries 4–9). Similarly, the CuBDC-catalyzed reactions yielded comparable results to HKUST-1, with slight variations in yield on the same base systems (Table 1, entries 13–18). A slight modification was explored by reducing the equivalent amount of one of the base variants to 0.5 and 0.2 equivalents. The results were consistent for both Cu-MOF catalysts, showing that a decrease in the amount of base led to a corresponding reduction in the reaction yield (Table 1, entries 10, 11 and 19, 20). Thus, we selected K_2_CO_3_ as the base for the next section of this research.

### 3.2 Effect of atmospheric conditions

It is evident that protodeboronation can occur under various atmospheric conditions, including air,^49^ O_2_,^50^ and an inert N_2_ atmosphere.^51^ Optimal conditions for copper salt-catalyzed protodeboronation of arylboronic acids were previously investigated by Liu *et al.* who reported that the reaction proceeds more efficiently under an O_2_ atmosphere compared to air or N_2_.^28^ The same study also highlighted that the active species in the copper-catalyzed protodeboronation reaction was Cu(ii), with oxygen playing a crucial role as an oxidant to regenerate the Cu(ii) species after protodeboronation occurred. To determine if these principles apply to our Cu-MOF catalysts, we conducted an atmospheric investigation using the previously established reaction model (Table 2). The atmosphere itself indeed played a role in facilitating the protodeboronation reaction when Cu-MOFs were used as the catalyst instead of copper salts. Higher yields were obtained under an oxygen atmosphere rather than in nitrogen (Table 2, entries 2 and 5 *vs.* 3 and 6). Interestingly, the results with an air atmosphere led to a higher protodeboronation product for CuBDC rather than HKUST-1 (Table 2, entries 1 and 4). This finding was in line with our previous work, where the use of HKUST-1 for arylboronic acid homocoupling in a normal atmosphere promoted the formation of its hydroxylation product, where Ar–B(OH)_2_ species is converted to Ar–OH, instead of the desired C–C coupling product. However, this side reaction could be prevented by conducting the reaction under an O_2_ atmosphere.^43^ This occurs due to the nature of HKUST-1, which can absorb moisture from the air into its structure and initiate the hydroxylation process.^52^ Additionally, the use of polar protic solvents such as ethanol and water can further promote this hydroxylation reaction. The same hydroxylation product was also observed in our reactions using the CuBDC catalyst, which was also present in the work by Puthiaraj *et al.*, who applied the same catalyst for aerobic arylboronic acid homocoupling.^53^ However, the amount of hydroxylation product found was smaller compared to that with HKUST-1. With these results, the HKUST-1 catalyst, K_2_CO_3_ base, and O_2_ atmosphere were selected as the optimized model for further experimentation.

**Table 2:** Atmosphere screening on the selected model of 4-*tert*-butylphenylboronic acid with K_2_CO_3_ as base^a^

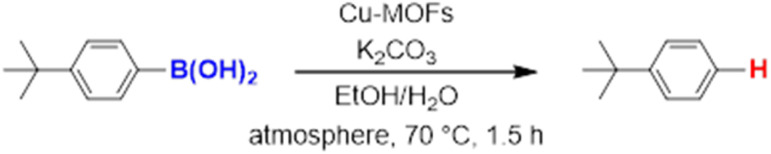			
Entry	Catalyst	Atmosphere	Yield^b^ (%)
1	HKUST-1	Air	65
2		O_2_	98
3		N_2_	54
4	CuBDC	Air	76
5		O_2_	95
6		N_2_	43

a Reaction conditions: 4-*tert*-butylphenylboronic acid (0.4 mmol), Cu-MOFs (10 mol%), K_2_CO_3_ (1 eq.) EtOH/H_2_O (1 mL/1 mL), 70 °C, 1.5 h.

b GC yields were reported with mesitylene as an internal standard.

### 3.3 Effect of substrate precursor

After optimizing the reaction conditions, we explored the substrate scope of arylboronic acids in the protodeboronation reaction. As shown in Table 3, the nature of the substituent—whether electron-donating or electron-withdrawing—significantly influences product formation. Notably, smaller substrates such as *p*-, *m*-, and *o*-tolylboronic acids (1a, 1b, 1c) exhibited lower yields compared to bulkier ones like *p*- and *o*-methoxyboronic acids (1d, 1e) (Table 3, entries 1–3 *vs.* 4 and 5). This trend is likely due to the porosity of HKUST-1, which can adsorb small protodeboronation products, as suggested by previous studies on its ability to capture small organic molecules such as benzene and toluene.^54–56^

**Table 3:** HKUST-1 catalyzed protodeboronation of arylboronic acids^a^

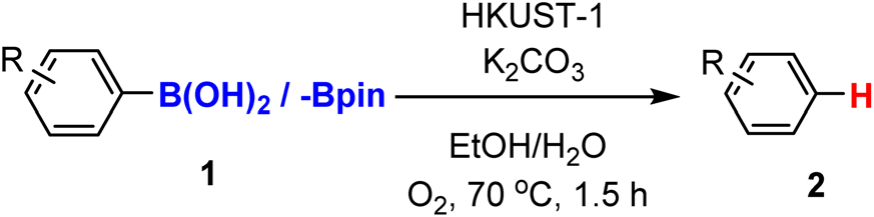				
Entry	Boronic reagents	Products	Yield^b^ (%)	Conversion^c^ (%)
1	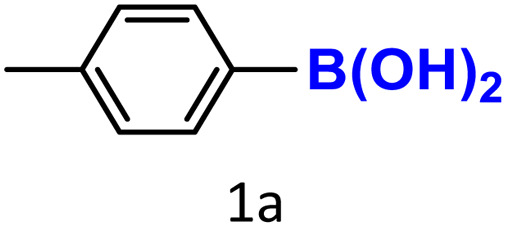	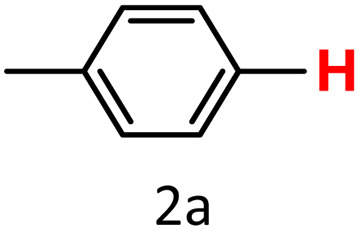	47(20)^d^	100
2	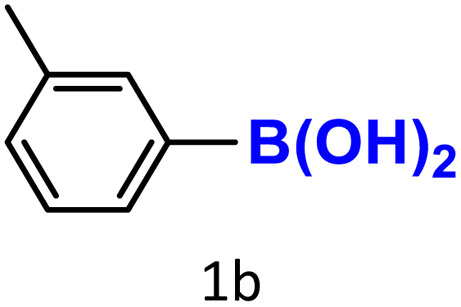	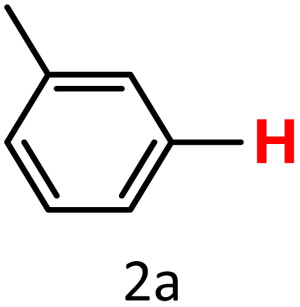	34(15)^d^	100
3	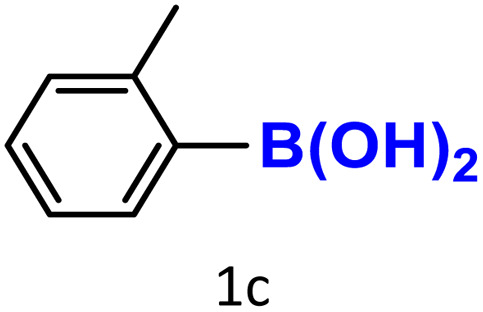	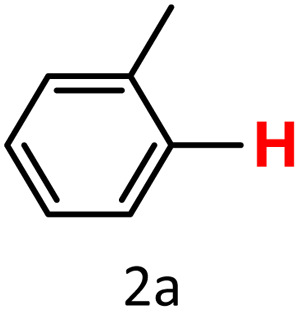	29	100
4	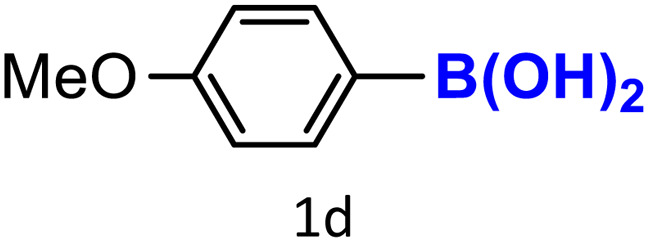	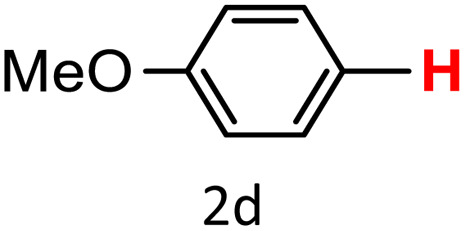	60	100
5	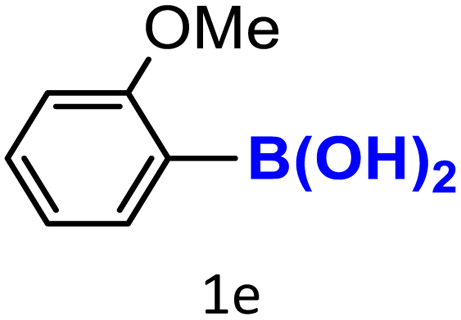	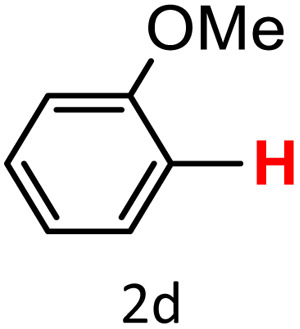	57	100
6^e^	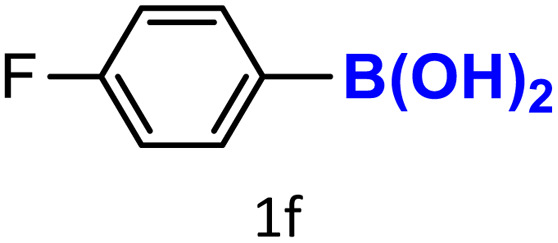	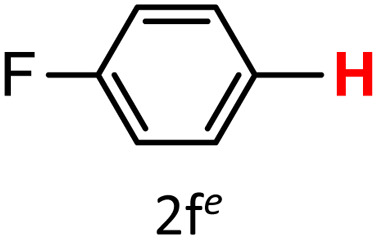	27(24)^d^	100
7	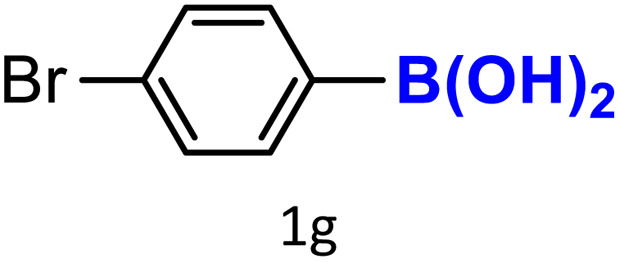	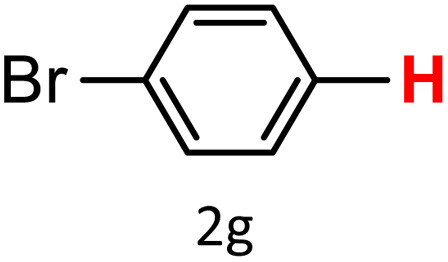	68	100
8	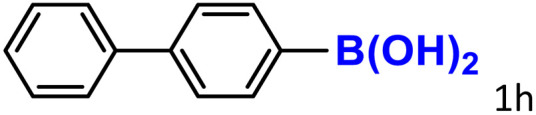	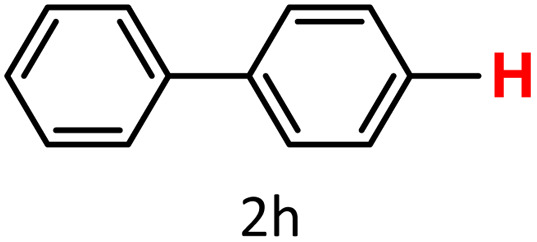	97(95)^f^	100
9	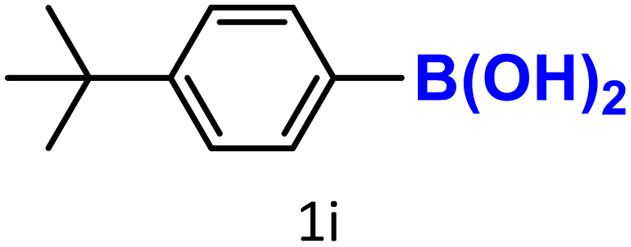	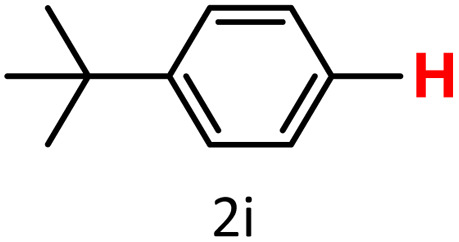	98(95)^d^	100
10	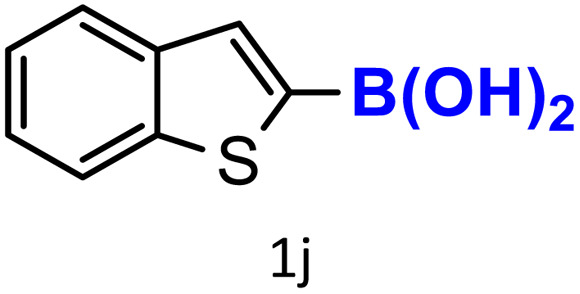	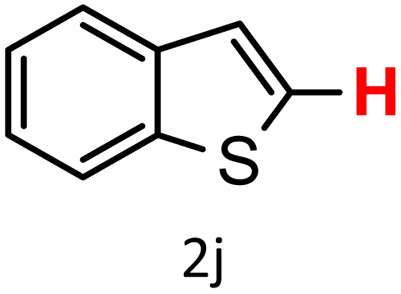	60(54)^f^	100
11	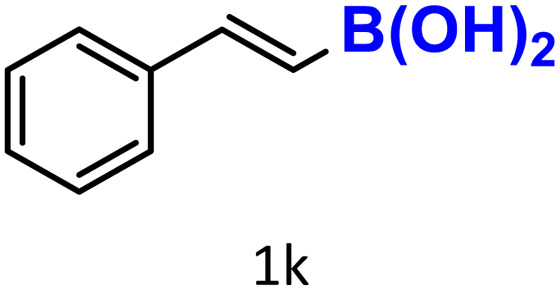	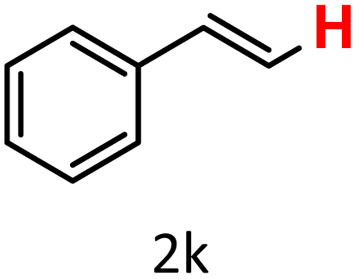	41	100
12	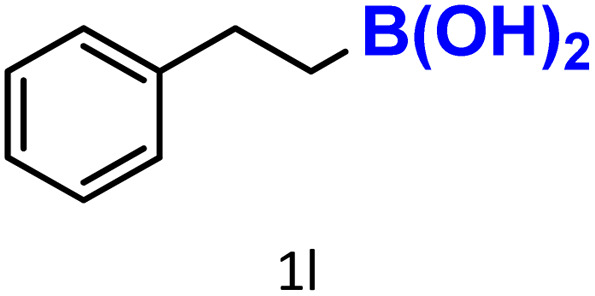	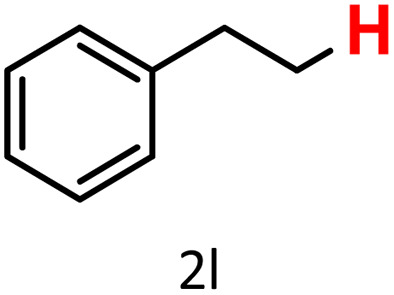	trace	100
13	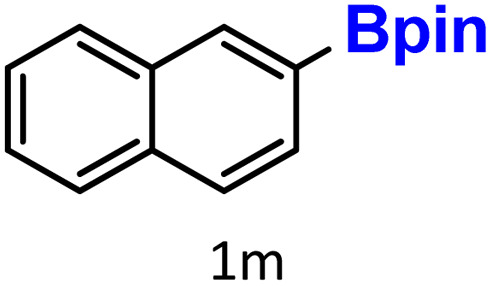	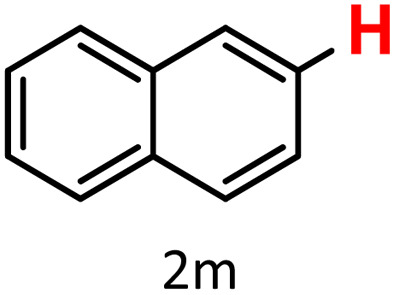	64(62)^f^	100
14	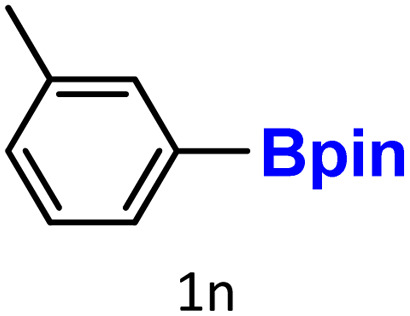	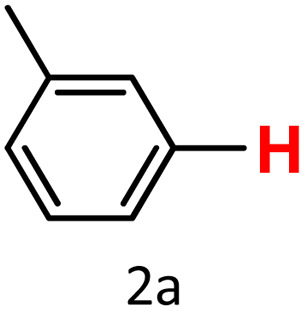	Trace	100

a Reaction conditions: arylboronic acid (0.4 mmol), HKUST-1 (10 mol%), K_2_CO_3_ (1 eq.) EtOH/H_2_O (1 mL/1 mL), O_2_, 70 °C, 1.5 h.

b GC yields were reported with mesitylene as an internal standard.

c Conversion based on remaining boronic reagents detected in GC.

d Using CuBDC (10 mol%).

e Due to high volatility of fluorobenzene (2f), the reaction proceeds at 40 °C.

f Isolated yield after column chromatography.

A similar effect was observed for bulkier substrates, including biphenyl-, 4-*tert*-butylphenyl-, and benzo[*b*]thien-2-ylboronic acids (1h, 1i, 1j), which yielded significant amounts of the corresponding protodeboronated products (Table 3, entries 8–10). Among electron-withdrawing substrates, *p*-fluorophenylboronic acid (1f) and *p*-bromophenylboronic acid (1g) also followed this trend, with the former yielding noticeably lower amounts, likely due to its smaller molecular size and lower reaction temperature (Table 3, entries 6 and 7).

To further investigate this behavior, we compared HKUST-1 with CuBDC as a catalyst for selected substrates (Table 3, entries 1, 2, 6, and 9). Interestingly, *p*-tolyl, *m*-tolyl, and *p*-fluorophenylboronic acids (1a, 1b, 1c) gave lower yields with CuBDC, while 4-*tert*-butylphenylboronic acid (1i) showed no significant difference between the two catalysts. An additional investigation was conducted to verify whether the small molecule was adsorbed into HKUST-1. To this end, 0.4 mmol of toluene was added as a substrate to 1 mL of ethanol containing 10 mol% of HKUST-1, and the mixture was stirred overnight. Gas chromatography (GC) analysis showed no detectable peak corresponding to toluene, confirming its adsorption into the MOF (Fig. S2.19). This supports the hypothesis that small molecules are more likely to be adsorbed within the MOF framework. Furthermore, steric effects play a role, as *o*-substituted substrates generally yielded lower amounts of product than their *p*- and *m*-substituted counterparts.

In addition to arylboronic acids, we examined heteroarylboronic acids and alkenyl- and alkyl-based boronic acids to expand the substrate scope. Benzo[*b*]thien-2-ylboronic acid (1j) underwent successful protodeboronation with HKUST-1 (Table 3, entry 10). Meanwhile, *trans*-2-phenylvinylboronic acid (1k) gave a moderate yield of the protodeboronated product (2k), whereas the protodeboronation of phenethylboronic acid (1l) was not detected, and interestingly it shows a minor hydroxylation product was observed. We have previously reported that HKUST-1 can promote the hydroxylation of electron-rich aryl–B(OH)_2_ under moist conditions in DMF, in the presence of O_2_ and notably in the absence of base.^43^ We believe that, in addition to minor formation of the hydroxylated byproduct of 1l, the majority of the resulting protodeboronation products may have remained adsorbed within the porous HKUST-1 framework, making them undetectable by standard GC-MS analysis.^52–54^ To verify this, we performed the reaction under standard conditions using ethylbenzene. After 1.5 hours, and with mesitylene as an internal standard, no ethylbenzene was detected by GC-MS, indicating its complete adsorption by HKUST-1.

Finally, we evaluated boronic acid pinacol esters (–Bpin) as substrates (Table 3, entries 13 and 14). A bulkier substrate, 2-naphthyl-Bpin (1m), underwent protodeboronation with moderate efficiency (2m). In contrast, a smaller substrate, *m*-tolyl-Bpin (1n), yielded only trace amounts of toluene (2a). Two key factors contribute to the absence of detectable protodeboronation products of *m*-tolyl-Bpin (1n) in the GC-MS analysis: (1) adsorption of toluene by HKUST-1, which reduces its volatility and recovery during analysis;^52–54^ (2) *in situ* formation of a boron–alkoxide adduct, such as [tolylBpin(OEt)]^−^, which is not amenable to GC-MS detection due to its ionic and non-volatile nature.

The latter has been demonstrated by Fernández and co-workers,^57^ who showed that –Bpin species can readily form Lewis acid–base adducts with alkoxide anions generated *in situ* from the combination of carbonate bases and alcohols. These adducts are typically non-volatile and thus remain undetected under standard GC-MS conditions.

Lloyd-Jones and co-workers have previously reported that the protodeboronation of electron-rich aryl boronic acids proceeds *via* a tetra-coordinate organoboronate intermediate formed through coordination with hydroxide.^58^ This is followed by a rate-limiting proton transfer from a water molecule, resulting in C–B bond cleavage. Therefore, in our case (Table 3, entry 14), the sluggish reactivity of *m*-tolyl-Bpin is likely halted at the stage of a stable boron–alkoxide adduct, which is undetectable by GC-MS.

To verify this, we conducted a control experiment in which *m*-tolyl-Bpin was treated with K_2_CO_3_ in an ethanol/water (1 : 1) mixture and stirred at 75 °C for 1.5 hours in the absence of the HKUST-1 catalyst. Under these conditions, and using a stoichiometric amount of mesitylene as an internal standard, neither the starting material nor the protodeboronation product was detected by GC-MS. It is also important to note that electron-rich aryl boronates generally exhibit greater resistance to protodeboronation than their electron-deficient counterparts. In a subsequent study,^59^ the same group investigated electron-deficient aryl boronates, such as 2,6-difluorophenyl derivatives, and found that Bpin esters undergo the slowest protodeboronation among various boronate esters, including those derived from catechol, ethylene glycol, 1,3-propanediol, 2-hydroxymethyl-2-methyl-1,3-propanediol, neopentylglycol, and 2,4-dimethylpentan-2,4-diol. Thus, the protodeboronation of aryl-Bpin derivatives is generally more difficult than that of their corresponding boronic acid (–B(OH)_2_) analogues.

**Scheme 3:**
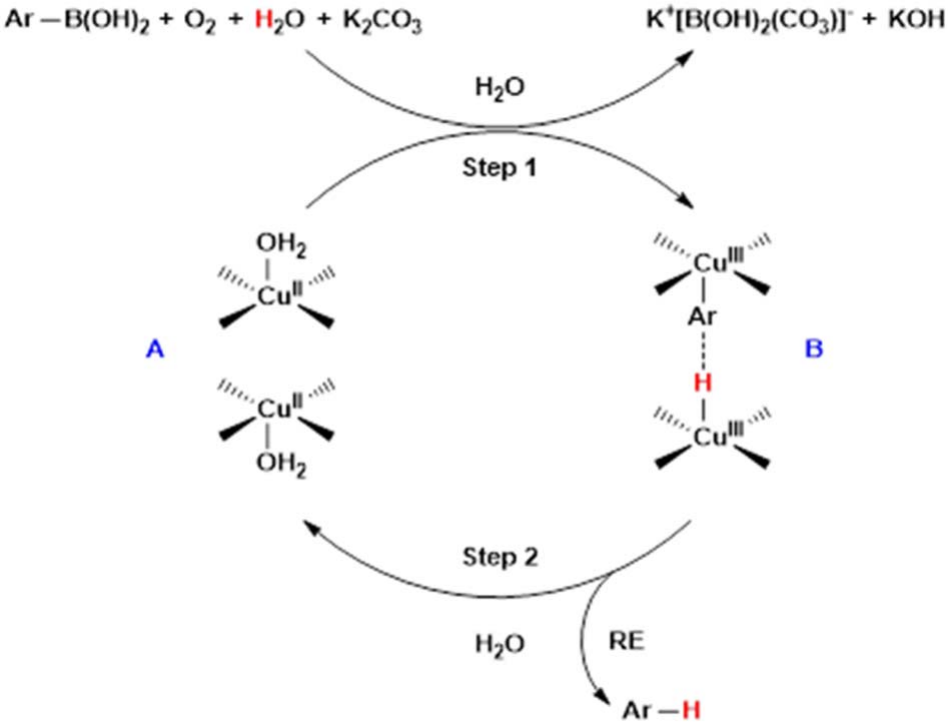
Proposed mechanism of Cu-MOF catalyzed protodeboronation.

We propose that this protodeboronation reaction proceeds *via* a bimetallic pathway (Scheme 3).^43,53,60^ Initially, oxidation of complex A with oxygen generates Cu(iii) species, which then react with Ar–B(OH)_2_, H_2_O, and K_2_CO_3_ to form intermediate B. This is followed by reductive elimination of the binuclear Cu(iii) complex, producing the protodeboronated product and regenerating the Cu(ii) catalyst in the presence of H_2_O, thereby sustaining the catalytic cycle.

### 3.4 Recyclability of the catalyst

**Fig. 1:**
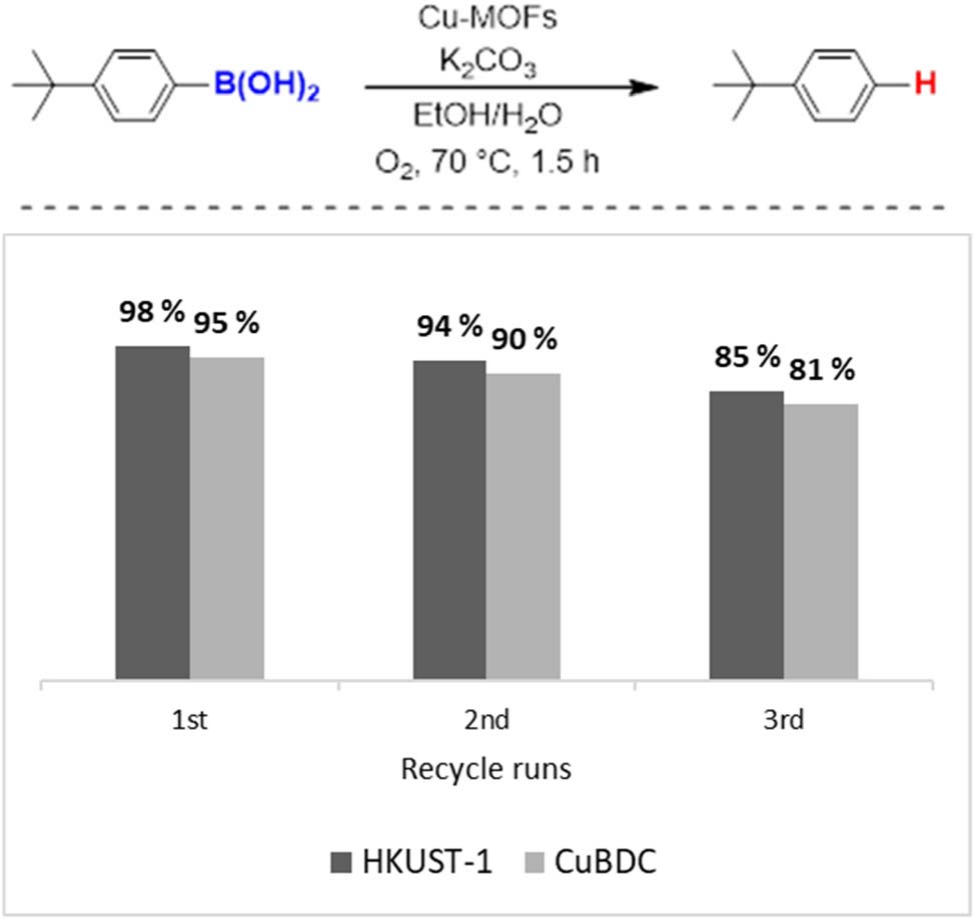
Recyclability studies of HKUST-1 and CuBDC in prodeboronation of arylboronic acid (reaction conditions: arylboronic acid (0.4 mmol), Cu-MOFs (10 mol%), K_2_CO_3_ (1 eq.) EtOH/H_2_O (1 mL/1 mL), O_2_, 70 °C, 1.5 h. GC yields were reported with mesitylene as an internal standard).

We evaluated the recyclability of both HKUST-1 and CuBDC, as these MOFs function as heterogeneous catalysts and can be reused for subsequent reaction runs.^43,53^ Interestingly, in a control reaction using 4-*tert*-butylphenylboronic acid (Fig. 1), both MOFs exhibited significant structural changes while still remaining recoverable from the reaction system. Fig. S3.1 shows that after use, HKUST-1 undergoes structural changes as indicated by the amorphous XRD pattern. In addition, the XRD pattern of CuBDC in Fig. S3.2 also shows structural changes after use and shows the characteristic peak of CuO. Nevertheless, the catalyst maintains a high protodeboronation yield for three consecutive runs, indicating that even the structural changed of the catalyst remains a viable copper source for catalysis.

To further investigate this, we conducted atomic absorption spectroscopy (AAS) analysis on the reaction mixture to assess potential copper leaching (see S4 SI). The analysis detected 0.081 ppm of copper, corresponding to only 0.0024% of the initial copper content, confirming that the MOF structure underwent structural changes, releasing a minimal amount of copper into the reaction medium. Nevertheless, the catalyst remained catalytically active for protodeboronation.

Structural changes of MOFs under basic conditions have been previously reported by Yuan *et al.* who observed that MOFs composed of high-valency metal ions and carboxylate-based ligands are particularly susceptible to degradation in alkaline environments.^61^ This instability arises from the low p*K*_a_ of carboxylic acids, which, despite forming strong coordination bonds with high-valency metal ions, render these MOFs highly stable in acidic conditions but less so in basic media. In contrast, MOFs with high p*K*_a_ ligands, such as azolate-based ligands paired with low-valency metal ions, are better suited for alkaline environments.

## 4 Conclusions

In summary, copper-based metal–organic frameworks (Cu-MOFs), including HKUST-1 and CuBDC, exhibit exceptional catalytic efficiency in the protodeboronation of arylboronic acids. Under optimized conditions with K_2_CO_3_ as a base and an O_2_ atmosphere, product yields reached up to 98% for HKUST-1 and 95% for CuBDC. Substrate variations revealed that steric and electronic effects significantly influence reaction outcomes. Larger substrates generally achieved higher conversions compared to smaller ones, likely due to the adsorption of smaller protodeboronation products within the porous frameworks of HKUST-1 and CuBDC. Both catalysts demonstrated promising recyclability over three cycles, even though structural changes in basic environments occurred. These findings highlight the potential of Cu-MOFs as versatile and efficient catalysts for protodeboronation, expanding their applications in sustainable catalysis.

## Author contributions

Yudha P. Budiman: conceptualization, formal analysis, supervision, validation, writing – original draft; Muhamad Rashifari: conceptualization, data curation, methodology, writing – original draft; Muhamad Diki Permana: data curation, visualization, writing – review & editing; Kansy Haikal: formal analysis; Iis I. Widyowati: data curation, formal analysis; Yessi Permana: formal analysis, validation; Ubed S. F. Arrozi: formal analysis, validation; Wirawan Ciptonugraha: formal analysis, validation; Tri Mayanti: supervision, validation; Allyn Pramudya Sulaeman: supervision, validation; Juliandri: supervision, validation; Witri Wahyu Lestari: conceptualization, validation.

## Conflicts of interest

The authors declare that there are no known competing financial interests or personal relationships capable of influencing the work reported in this paper.

## Supplementary Material

RA-015-D5RA04172D-s001

## Data Availability

The data supporting this article have been included as part of the SI. See DOI: https://doi.org/10.1039/d5ra04172d.
