# Supplementary material for: Catalytic efficiency of Cu-MOFs: HKUST-1 and CuBDC for the protodeboronation of aryl boronic acids
Source: RSC Adv. 2025 Aug 21;15(36):29453–61. doi: 10.1039/d5ra04172d (PMC12377164; doi:10.1039/d5ra04172d)
Supplement: RA-015-D5RA04172D-s001 [file RA-015-D5RA04172D-s001.pdf]

Supplementary Information (SI) for RSC Advances.
This journal is © The Royal Society of Chemistry 2025

Supporting Information for

Catalytic Efficiency of Cu-MOFs: HKUST-1 and CuBDC for the Protodeboronation of Aryl Boronic Acids

Yudha P. Budiman,[a,þ,*] Muhamad Rashifari,[a,þ] Muhamad Diki Permana,[b,c] Kansy Haikal,[a] Iis I. Widyowati,[a] Yessi Permana,[d] Ubed S. F. Arrozi,[e] Wirawan Ciptonugraha,[f] Tri Mayanti,[a] Allyn P. Sulaeman,[a] Juliandri,[a] Witri Wahyu Lestari[f]

[a]Department of Chemistry, Faculty of Mathematics and Natural Sciences, Universitas Padjadjaran, 45363 Sumedang, Indonesia

[b]Special Educational Program for Green Energy Conversion Science and Technology, Integrated Graduate School of Medicine, Engineering, and Agricultural Sciences, University of Yamanashi, Kofu 400-8511, Japan

[c]Center for Crystal Science and Technology, University of Yamanashi, Kofu 400-8511, Japan

[d]Inorganic and Physical Chemistry Research Division, Faculty of Mathematics and Natural Sciences, Institut Teknologi Bandung, Bandung 40132, Indonesia

[e]Department of Chemistry, Faculty of Mathematics and Natural Sciences, State University of Malang, 65145, Malang, Indonesia

[f]Department of Chemistry, Faculty of Mathematics and Natural Sciences, Universitas Sebelas Maret, Surakarta, 57126, Indonesia,

*Corresponding author: y.p.budiman@unpad.ac.id

S1. General Information

Unless otherwise noted, all reagents were purchased from Sigma-Aldrich were checked for purity by GC-MS and used as received. Absolute ethanol (Sigma-Aldrich, 99.8%) was used as received. The removal of solvent was performed by an extraction method in a mixture of brine water (2 mL) and ethyl acetate (2 mL) three times.

High-angle X-ray diffraction patterns were obtained on a Rigaku Miniflex 600 D/teX Ultra diffractometer with Cu Kα radiation. Infrared spectra were recorded on a Nicolet 380 FT-IR spectrometer as solids, using an ATR unit, and are reported in cm^{-1}. GC-MS analyses were performed using an Agilent 7890A gas chromatograph (column: DB-5MS 5% phenylmethylsiloxane, 30 m, Ø 0.25 mm, film 0.25 µm; *injector: 200 °C; oven: 40 °C (2 min), 40 °C to 280 °C (20 °C min^{-1}) (5 min) *; carrier gas: He (1 mL min^{-1}) equipped with an Agilent 5977B GC/MSD operating in EI mode). Copper atomic absorption spectrometry were conducted on a Perkin Elmer AAnalyst 400 AA with Series N30501XX Copper (Cu) Lumina hollow cathode lamp.

All NMR spectra were recorded at 298 K using an Agilent DD2 (^{1}H-NMR, 500 MHz; ^{13}C{^{1}H}-NMR, 125 MHz). ^{1}H-NMR chemical shifts are reported relative to TMS and were referenced via residual proton resonances of the corresponding deuterated solvent ($CDCl_3$: 7.24 ppm), ^{13}C{^{1}H}-NMR spectra are reported relative to TMS via the carbon signals of the deuterated solvent ($CDCl_3$: 77.16 ppm

S1.1 Synthesis of toluene (2a) from *p*-tolylboronic acid (1a)

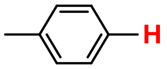

Compound **2a** was synthesized following the general procedure and using the following chemicals and conditions: *p*-tolylboronic acid (54.3 mg, 0.4 mmol), HKUST-1 (26.4 mg, 0.04 mmol), K_2CO_3 (55.3 mg, 0.4 mmol), mesitylene (56 μL, 0.4 mmol), EtOH/H_2O (1 mL : 1 mL), O_2 atmosphere, 70 °C, and 1.5 h.

GCMS: [t = 3.630 min] m/z: 91 $[M]^+$.

This compound is known.[1]

S1.2 Synthesis of toluene (2a) from *m*-tolylboronic acid (1b)

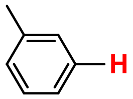

Compound **2a** was synthesized following the general procedure and using the following chemicals and conditions: *m*-tolylboronic acid (54.3 mg, 0.4 mmol), HKUST-1 (26.4 mg, 0.04 mmol), K_2CO_3 (55.3 mg, 0.4 mmol), mesitylene (56 μL, 0.4 mmol), EtOH/H_2O (1 mL : 1 mL), O_2 atmosphere, 70 °C, and 1.5 h.

GCMS: [t = 3.625 min] m/z: 91 $[M]^+$.

This compound is known.[1]

S1.3 Synthesis of toluene (2a) from *o*-tolylboronic acid (1c)

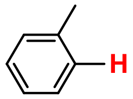

Compound **2a** was synthesized following the general procedure and using the following chemicals and conditions: *o*-tolylboronic acid (54.3 mg, 0.4 mmol), HKUST-1 (26.4 mg,

0.04 mmol), K_2CO_3 (55.3 mg, 0.4 mmol), mesitylene (56 μL, 0.4 mmol), EtOH/H_2O (1 mL : 1 mL), O_2 atmosphere, 70 °C, and 1.5 h.

GCMS: [t = 3.633 min] m/z: 91 $[M]^+$.

This compound is known.[1]

S1.4 Synthesis of anisole (2d) from *p*-methoxyphenylboronic acid (1d)

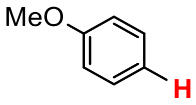

Compound **2d** was synthesized following the general procedure and using the following chemicals and conditions: *p*-methoxyphenylboronic acid (60.8 mg, 0.4 mmol), HKUST-1 (26.4 mg, 0.04 mmol), K_2CO_3 (55.3 mg, 0.4 mmol), mesitylene (56 μL, 0.4 mmol), EtOH/H_2O (1 mL : 1 mL), O_2 atmosphere, 70 °C, and 1.5 h.

GCMS: [t = 5.353 min] m/z: 109 $[M]^+$.

This compound is known.[1]

S1.5 Synthesis of anisole (2d) from *o*-methoxyphenylboronic acid (1e)

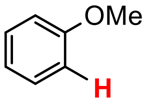

Compound **2d** was synthesized following the general procedure and using the following chemicals and conditions: *o*-methoxyphenylboronic acid (60.8 mg, 0.4 mmol), HKUST-1 (26.4 mg, 0.04 mmol), K_2CO_3 (55.3 mg, 0.4 mmol), mesitylene (56 μL, 0.4 mmol), EtOH/H_2O (1 mL : 1 mL), O_2 atmosphere, 70 °C, and 1.5 h.

GCMS: [t = 5.346 min] m/z: 109 $[M]^+$.

This compound is known.[1]

S1.6 Synthesis of fluorobenzene (2f)

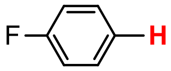

Compound **2f** was synthesized following the general procedure and using the following chemicals and conditions: *p*-fluorophenylboronic acid (56.0 mg, 0.4 mmol), HKUST-1 (26.4 mg, 0.04 mmol), K_2CO_3 (55.3 mg, 0.4 mmol), mesitylene (56 µL, 0.4 mmol), EtOH/H_2O (1 mL : 1 mL), O_2 atmosphere, 40 °C, and 1.5 h.

GCMS: [t = 2.626 min] m/z: 96 $[M]^+$.

This compound is known.[2]

S1.7 Synthesis of bromobenzene (2g)

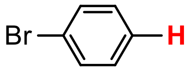

Compound **2g** was synthesized following the general procedure and using the following chemicals and conditions: *p*-bromophenylboronic acid (80.3 mg, 0.4 mmol), HKUST-1 (26.4 mg, 0.04 mmol), K_2CO_3 (55.3 mg, 0.4 mmol), mesitylene (56 µL, 0.4 mmol), EtOH/H_2O (1 mL : 1 mL), O_2 atmosphere, 70 °C, and 1.5 h.

GCMS: [t = 5.518 min] m/z: 156 $[M]^+$.

This compound is known.[2]

S1.8 Synthesis of biphenyl (2h)

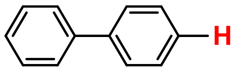

Compound **2h** was synthesized following the general procedure and using the following chemicals and conditions: biphenylboronic acid (79.2 mg, 0.4 mmol), HKUST-1 (26.4

mg, 0.04 mmol), K_2CO_3 (55.3 mg, 0.4 mmol), mesitylene (56 µL, 0.4 mmol), EtOH/H_2O (1 mL : 1 mL), O_2 atmosphere, 70 °C, and 1.5 h.

GCMS: [t = 9.287 min] m/z: 155 $[M]^+$.

^{1}H NMR (500 MHz, CDCl3) δ = 7.64–7.63 (*m*, 4H; Ar-H), 7.48 (*t*, 4H, $^3J_{H,H}$ = 8 Hz; Ar-H), 7.39 (*t*, 2H, $^3J_{H,H}$ = 7 Hz; Ar-H); **^{13}C{^{1}H} NMR** (125 MHz, CDCl3) δ = 141.4, 128.9, 127.4, 127.3

This compound is known.[2]

S1.9 Synthesis of *tert*-butylbenzene (2i)

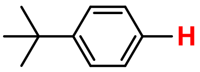

Compound **2i** was synthesized following the general procedure and using the following chemicals and conditions: *tert*-butylphenylboronic acid (71.2 mg, 0.4 mmol), HKUST-1 (26.4 mg, 0.04 mmol), K_2CO_3 (55.3 mg, 0.4 mmol), mesitylene (56 µL, 0.4 mmol), EtOH/H_2O (1 mL : 1 mL), O_2 atmosphere, 70 °C, and 1.5 h.

GCMS: [t = 6.091 min] m/z: 134 $[M]^+$.

This compound is known.[2]

S1.10 Synthesis of benzo[*b*]thiophene (2j)

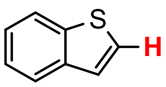

Compound **2j** was synthesized following the general procedure and using the following chemicals and conditions: benzo[*b*]thien-2-ylboronic acid (71.2 mg, 0.4 mmol), HKUST-1 (26.4 mg, 0.04 mmol), K_2CO_3 (55.3 mg, 0.4 mmol), mesitylene (56 µL, 0.4 mmol), EtOH/H_2O (1 mL : 1 mL), O_2 atmosphere, 70 °C, and 1.5 h. Due to strong interactions

between sulfur-containing heterocycles such as benzo[*b*]thiophene (**2j**) with silica gel during column chromatography, elution becomes challenging, resulting in a low isolated yield that is insufficient for NMR measurement.

GCMS: [t = 7.921 min] m/z: 134 [M]$^+$.

This compound is known.[3]

S1.11 Synthesis of styrene (2k)

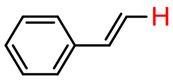

Compound **2k** was synthesized following the general procedure and using the following chemicals and conditions: trans-2-Phenylvinylboronic acid (29.6 mg, 0.2 mmol), HKUST-1 (13.2 mg, 0.02 mmol), K_2CO_3 (27.6 mg, 0.2 mmol), mesitylene (28 µL, 0.2 mmol), EtOH/H_2O (1 mL : 1 mL), O_2 atmosphere, 70 °C, and 1.5 h.

GCMS: [t = 4.946 min] m/z: 104 [M]$^+$.

This compound is known.[4]

S1.12 Synthesis of ethylbenzene (2l)

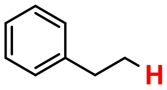

Compound **2l** was synthesized following the general procedure and using the following chemicals and conditions: trans-2-Phenylvinylboronic acid (30 mg, 0.2 mmol), HKUST-1 (13.2 mg, 0.02 mmol), K_2CO_3 (27.6 mg, 0.2 mmol), mesitylene (28 µL, 0.2 mmol), EtOH/H_2O (1 mL : 1 mL), O_2 atmosphere, 70 °C, and 1.5 h.

This compound is known.[5]

S1.13 Synthesis of naphthalene (2m)

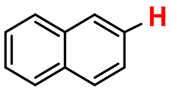

Compound **2m** was synthesized following the general procedure and using the following chemicals and conditions: 4,4,5,5-tetramethyl-2-(naphthalen-2-yl)-1,3,2-dioxaborolane (50.82 mg, 0.2 mmol), HKUST-1 (13.2 mg, 0.02 mmol), K_2CO_3 (27.6 mg, 0.2 mmol), mesitylene (28 µL, 0.2 mmol), EtOH/H_2O (1 mL : 1 mL), O_2 atmosphere, 70 °C, and 1.5 h.

GCMS: [t = 7.759 min] m/z: 128 $[M]^+$.**^{1}H NMR** (500 MHz, $CDCl_3$) δ = 3.10 (*q*, 4H, $^3J_{H,H}$ = 3 Hz), 7.52 (*d*, 4H, $^3J_{H,H}$ = 3 Hz); **^{13}C NMR** (125 MHz, $CDCl_3$) δ = 133,5, 128.0, 125.9; **GCMS**: [t = 7.935 min] m/z: 134 $[M]^+$.

This compound is known.[2]

S1.14 Synthesis of toluene (2a) from 4,4,5,5-tetramethyl-2-(*m*-tolyl)-1,3,2-dioxaborolane (1n)

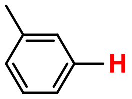

Compound **2n** was synthesized following the general procedure and using the following chemicals and conditions: 4,4,5,5-tetramethyl-2-(*m*-tolyl)-1,3,2-dioxaborolane (43.6 mg, 0.2 mmol), HKUST-1 (13.2 mg, 0.02 mmol), K_2CO_3 (27.6 mg, 0.2 mmol), mesitylene (28 µL, 0.2 mmol), EtOH/H_2O (1 mL : 1 mL), O_2 atmosphere, 70 °C, and 1.5 h.

GCMS: [t = 3.633 min] m/z: 91 $[M]^+$.

This compound is known.[1]

S2. GC-MS and ^{1}H, $^{13}C\{^{1}H\}$ NMR Spectra

S2.1 GC–MS spectrum of the reaction described in Table 3, entry 1, with mesitylene as the internal standard.

```
File       :D:\Lab Sentral\Data\2022\Yudha Reserch Grup\2024\AR 27.D
Operator   :
Acquired   : 15 Nov 2024  17:45     using AcqMethod Umi.M
Instrument :    GCMS
Sample Name:
Misc Info  :
Vial Number: 0
```

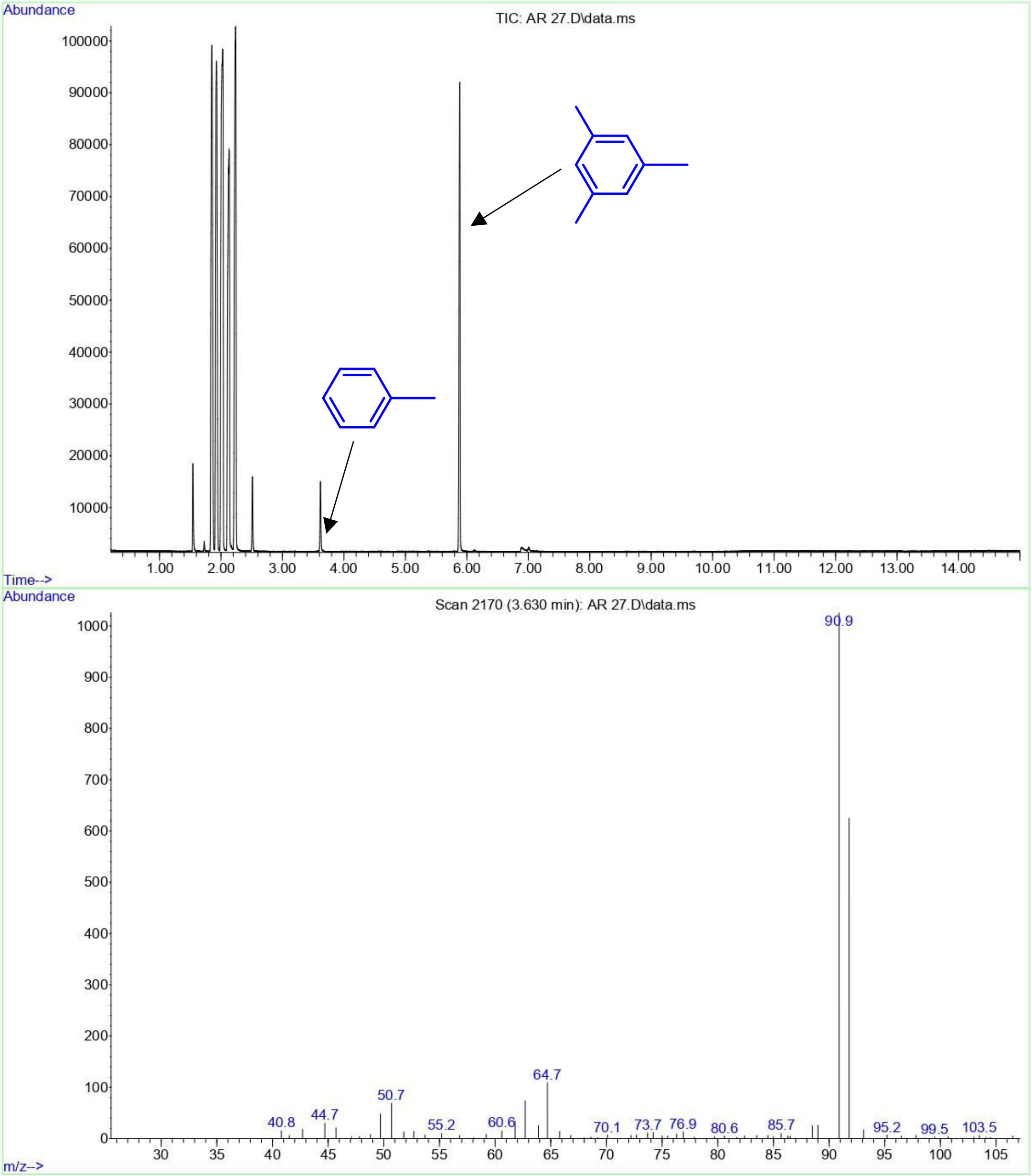

S2.2 GC–MS spectrum of the reaction described in Table 3, entry 2, with mesitylene as the internal standard.

```
File       :D:\Lab Sentral\Data\2022\Yudha Reserch Grup\2024\AR 29.D
Operator   :
Acquired   : 28 Nov 2024  07:46     using AcqMethod Umi.M
Instrument :    GCMS
Sample Name:
Misc Info  :
Vial Number: 0
```

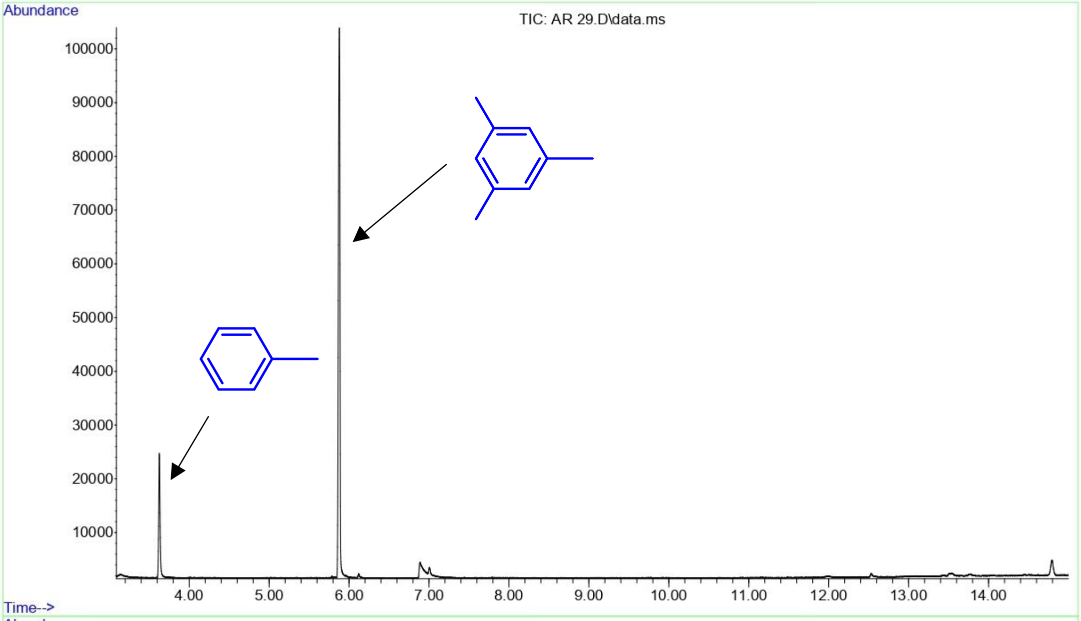

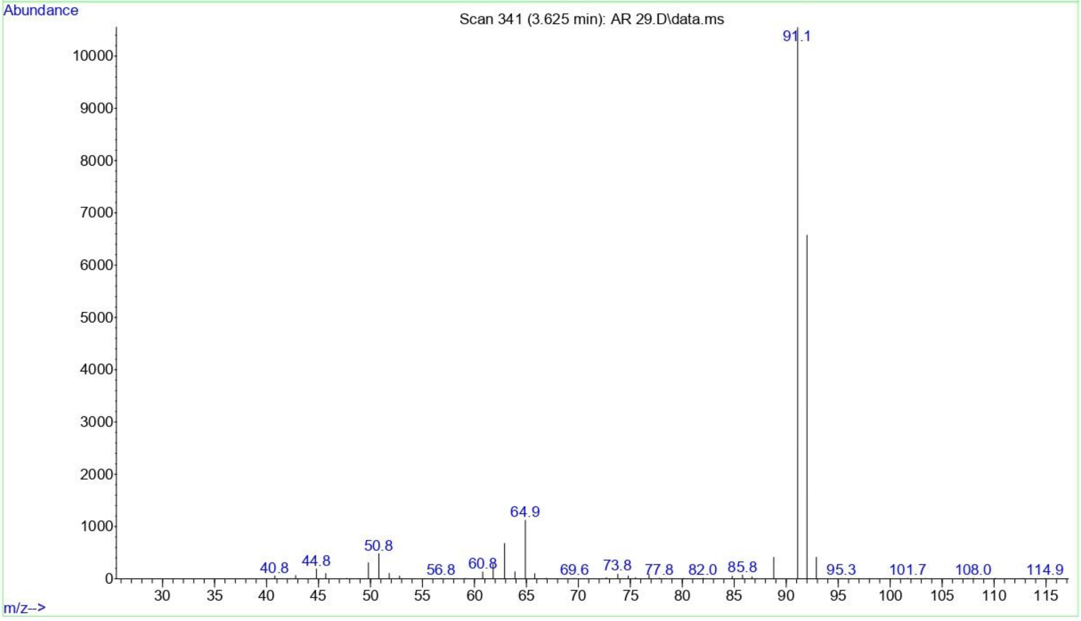

S2.3 GC–MS spectrum of the reaction described in Table 3, entry 3, with mesitylene as the internal standard.

```
File       :D:\Lab Sentral\Data\2022\Yudha Reserch Grup\2024\AR 30.D
Operator   :
Acquired   : 28 Nov 2024  08:25     using AcqMethod Umi.M
Instrument :    GCMS
Sample Name:
Misc Info  :
Vial Number: 0
```

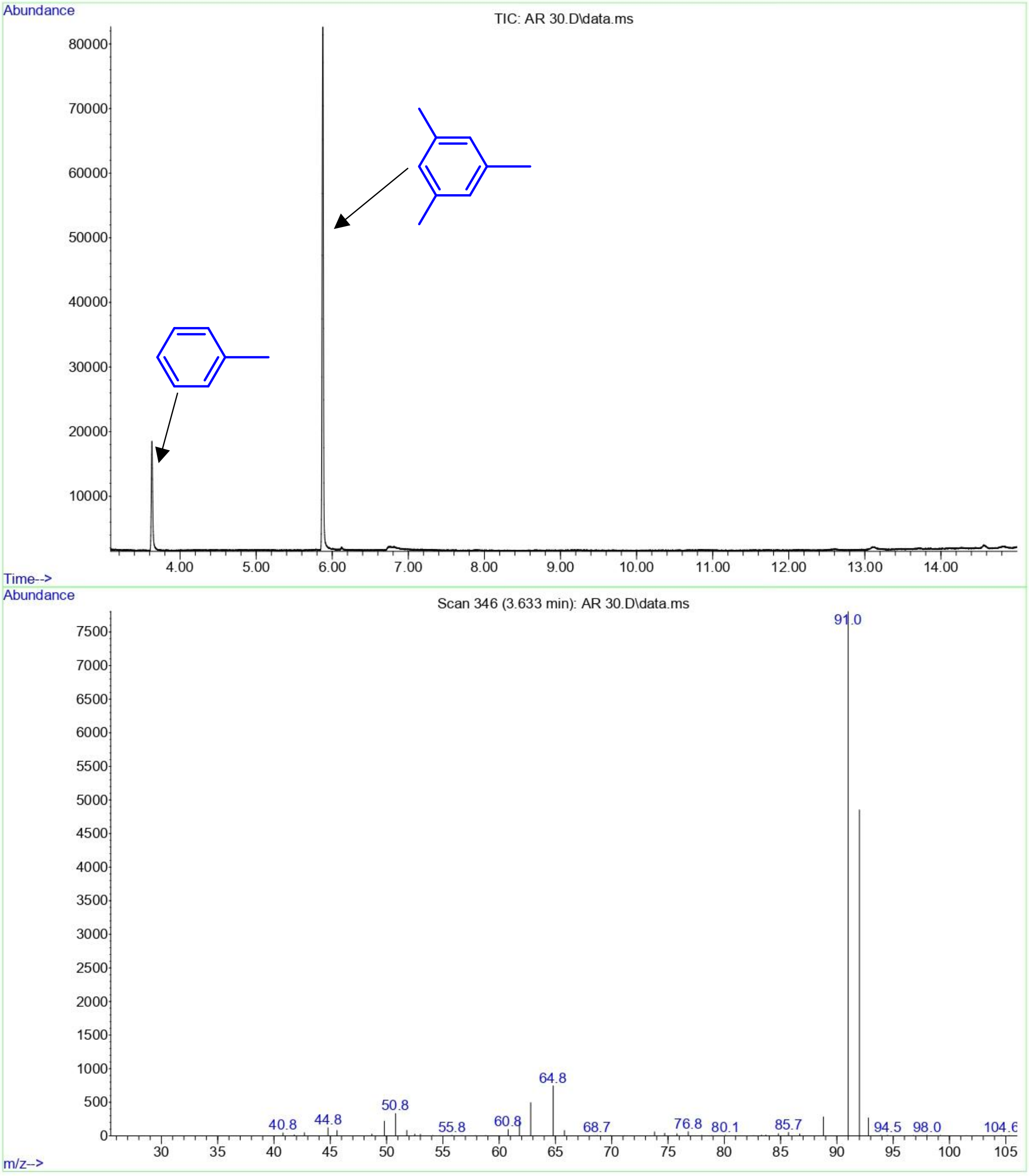

S2.4 GC–MS spectrum of the reaction described in Table 3, entry 4, with mesitylene as the internal standard.

```
File       :D:\Lab Sentral\Data\2022\Yudha Reserch Grup\2024\AR 23.D
Operator   :
Acquired   : 15 Nov 2024  16:09     using AcqMethod Umi.M
Instrument :    GCMS
Sample Name:
Misc Info  :
Vial Number: 0
```

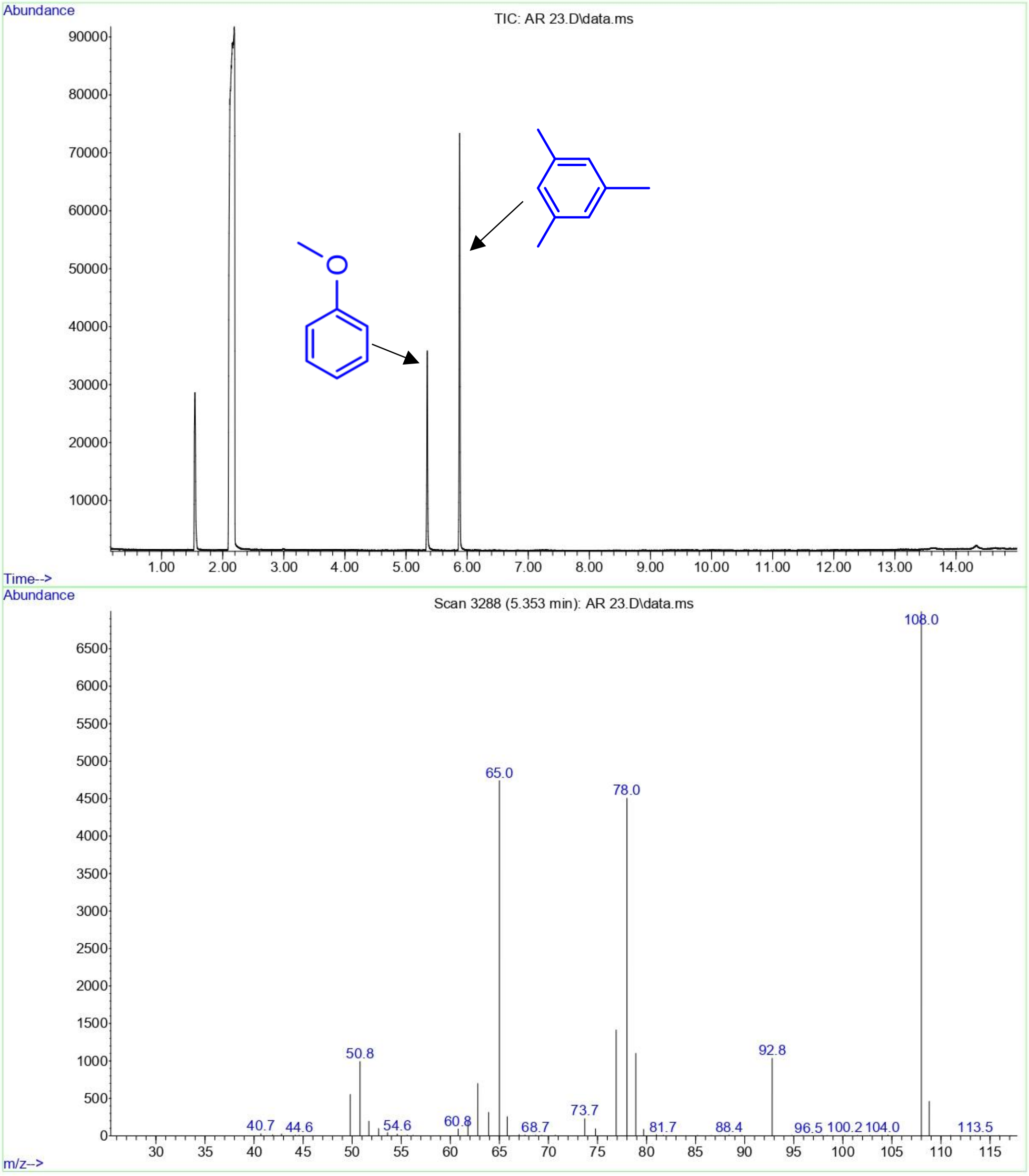

S2.5 GC–MS spectrum of the reaction described in Table 3, entry 5, with mesitylene as the internal standard.

```
File       :D:\Lab Sentral\Data\2022\Yudha Reserch Grup\2024\AR.D
Operator   :
Acquired   : 15 Nov 2024  14:31     using AcqMethod Umi.M
Instrument :    GCMS
Sample Name:
Misc Info  :
Vial Number: 0
```

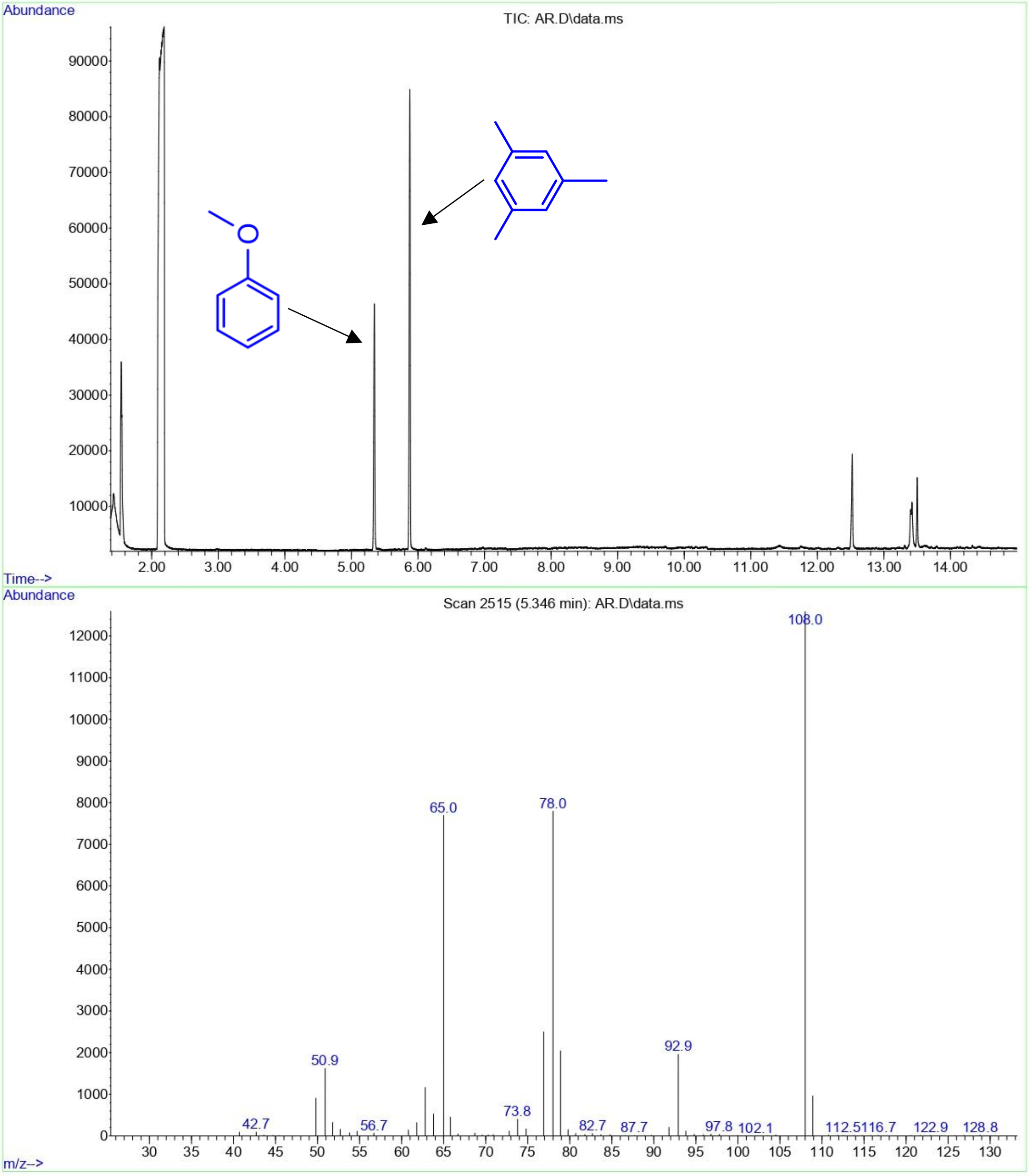

S2.6 GC–MS spectrum of the reaction described in Table 3, entry 6, with mesitylene as the internal standard.

```
File       :D:\Lab Sentral\Data\2022\Yudha Reserch Grup\2024\AR 26.D
Operator   :
Acquired   : 15 Nov 2024  16:58     using AcqMethod Umi.M
Instrument :    GCMS
Sample Name:
Misc Info  :
Vial Number: 0
```

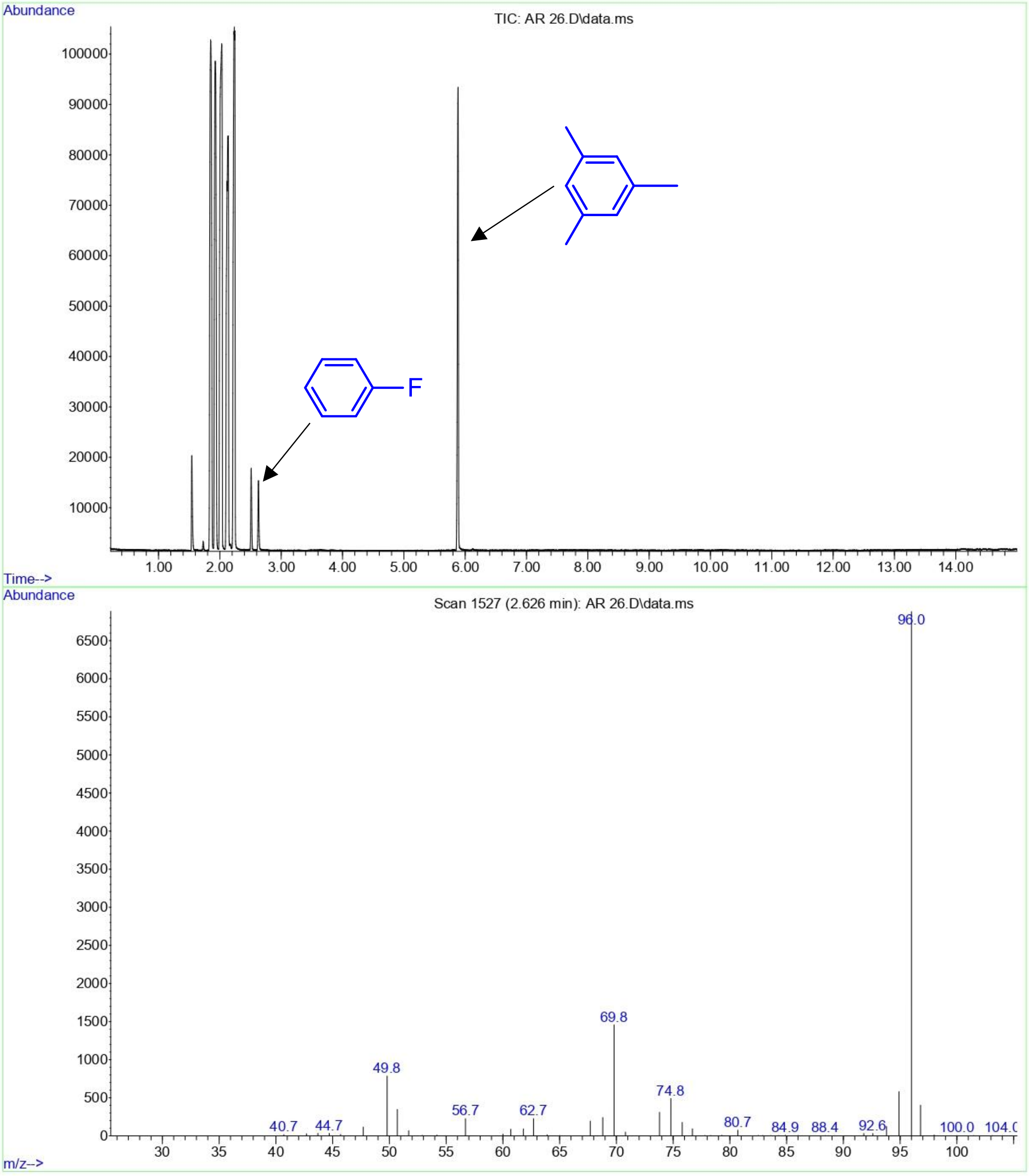

S2.7 GC–MS spectrum of the reaction described in Table 3, entry 7, with mesitylene as the internal standard.

```
File       :D:\Lab Sentral\Data\2022\Yudha Reserch Grup\2024\AR 25.D
Operator   :
Acquired   : 15 Nov 2024  16:33     using AcqMethod Umi.M
Instrument :    GCMS
Sample Name:
Misc Info  :
Vial Number: 0
```

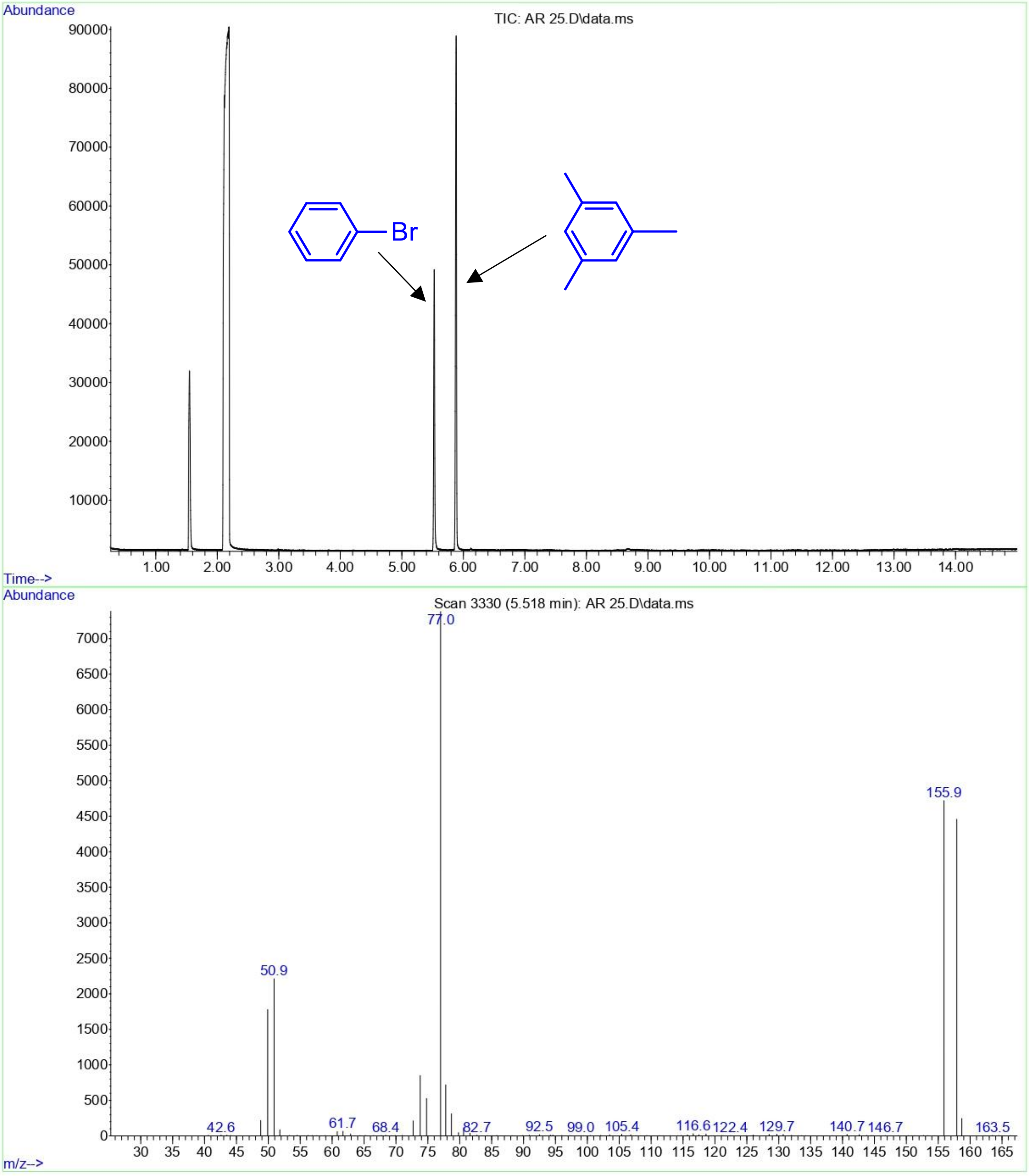

S2.8 GC–MS spectrum of the reaction described in Table 3, entry 8, with mesitylene as the internal standard.

```
File       :D:\Lab Sentral\Data\2022\Yudha Reserch Grup\2024\AR 14.D
Operator   :
Acquired   : 07 Nov 2024  13:56     using AcqMethod Umi.M
Instrument :    GCMS
Sample Name:
Misc Info  :
Vial Number: 0
```

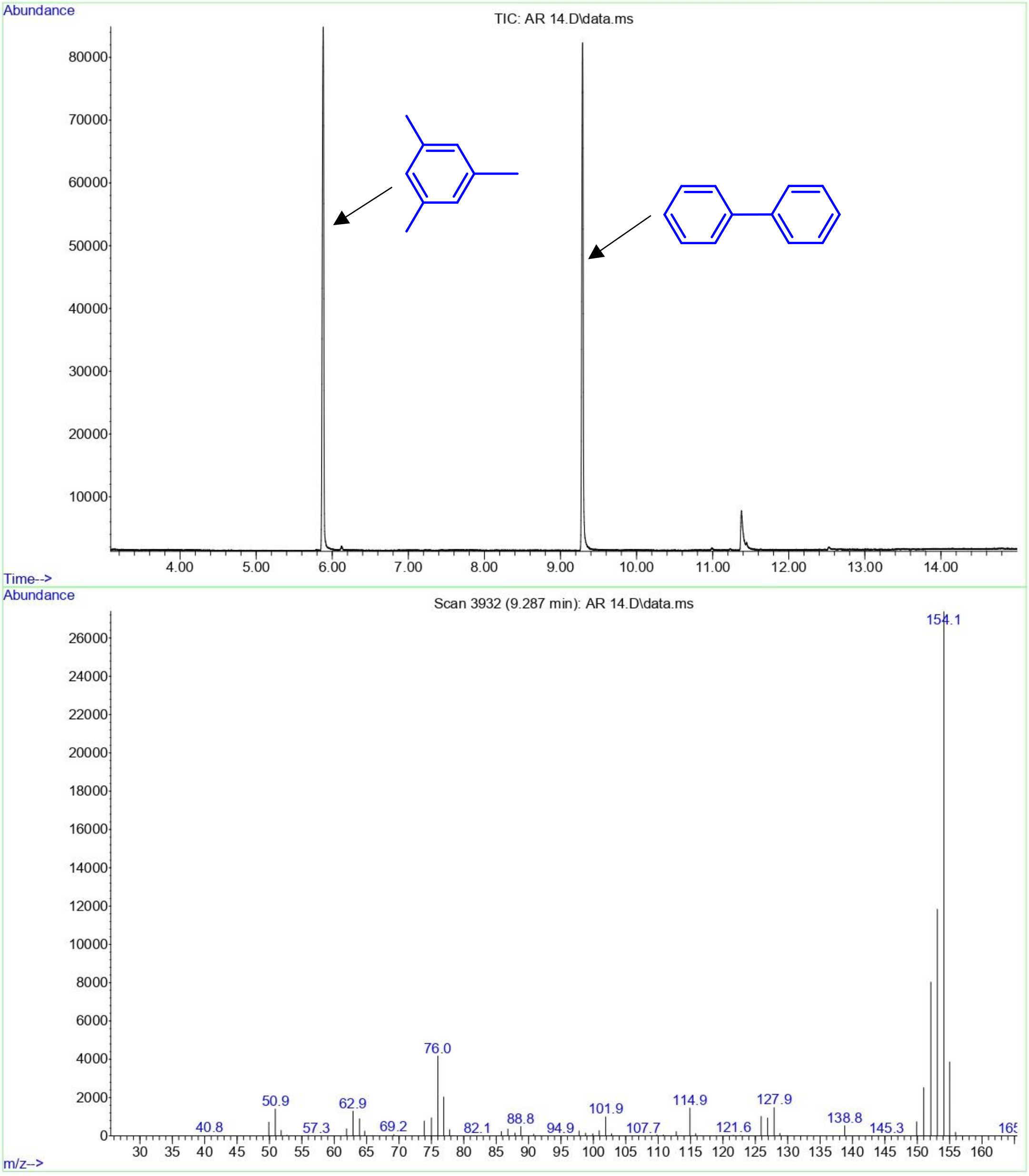

S2.9 ^{1}H NMR spectrum of **2h** (500 MHz, $CDCl_3$)

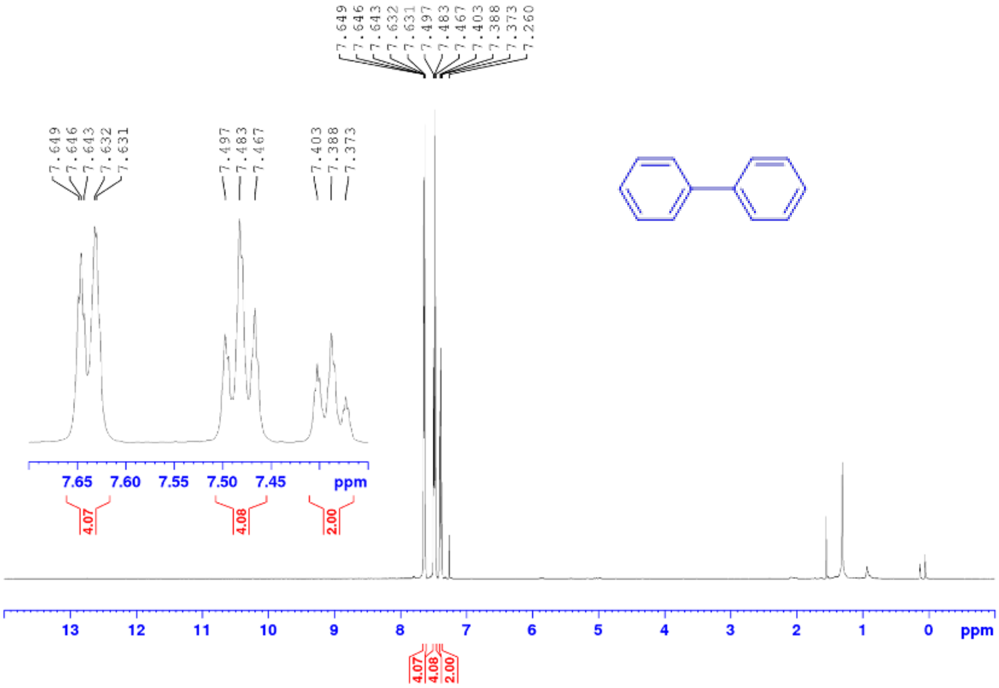

S2.10 ^{13}C{^{1}H} NMR spectrum of **2h** (125 MHz, $CDCl_3$)

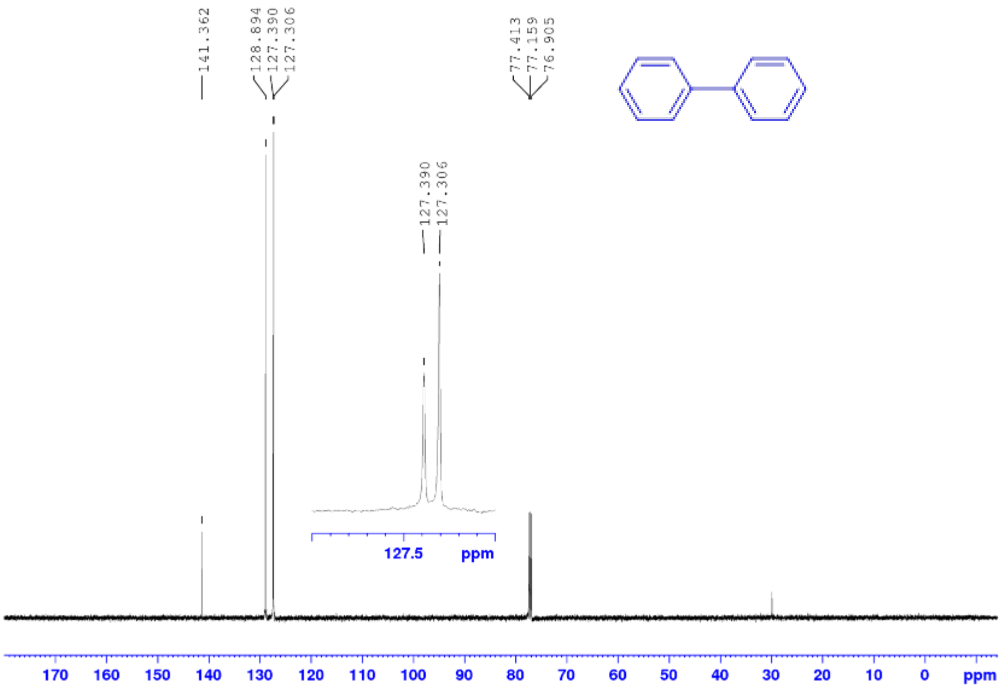

S2.11 GC–MS spectrum of the reaction described in Table 3, entry 9, with mesitylene as the internal standard.

```
File       :D:\Lab Sentral\Data\2022\Yudha Reserch Grup\2024\AR 06 B.D
Operator   :
Acquired   : 19 Aug 2024  08:40     using AcqMethod Umi.M
Instrument :    GCMS
Sample Name:
Misc Info  :
Vial Number: 0
```

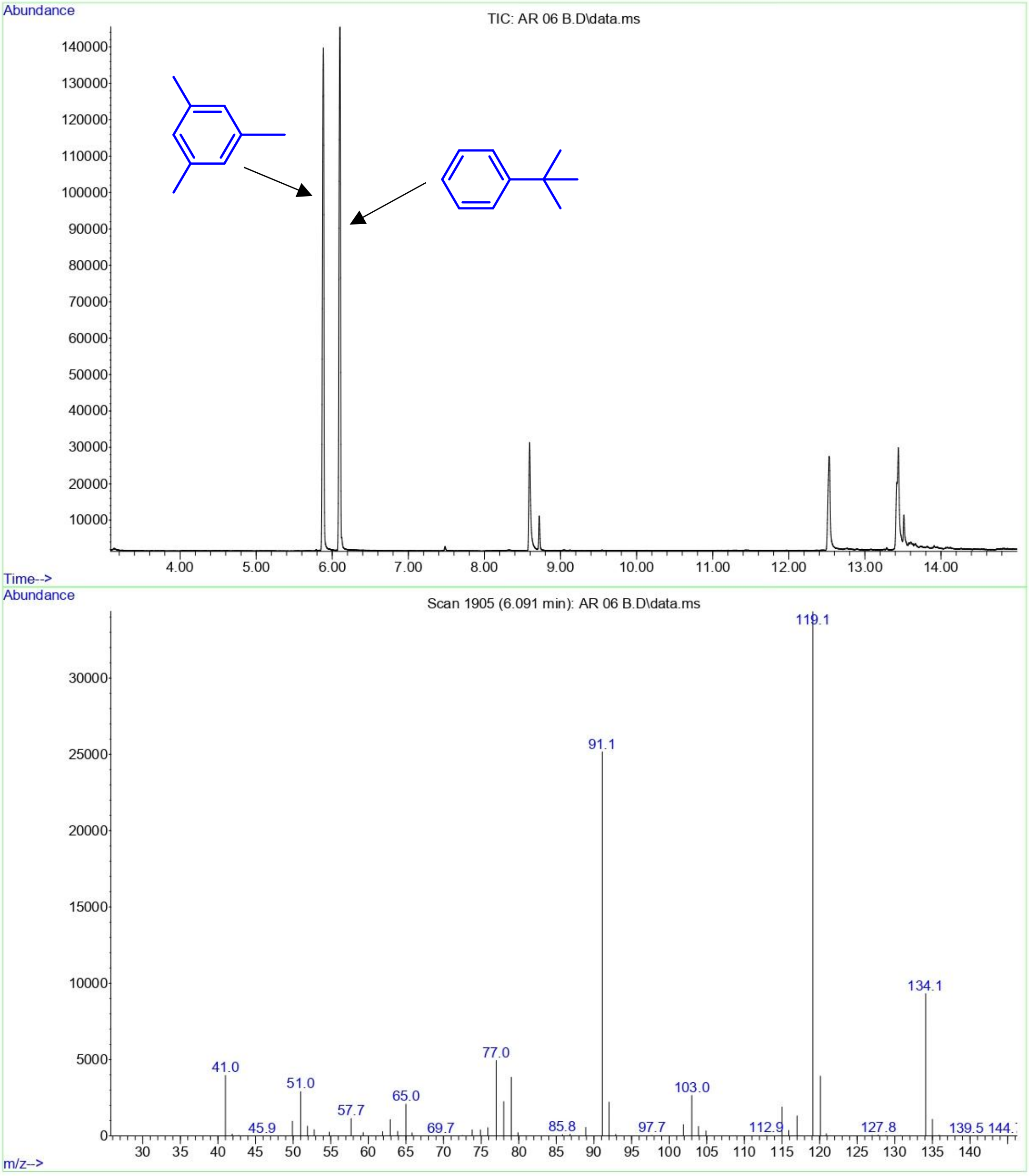

S2.12 GC–MS spectrum of the reaction described in Table 3, entry 10, with mesitylene as the internal standard.

```
File       :D:\Lab Sentral\Data\2022\Yudha Reserch Grup\2024\AR 21.D
Operator   :
Acquired   : 12 Nov 2024  08:09     using AcqMethod Umi.M
Instrument :    GCMS
Sample Name:
Misc Info  :
Vial Number: 0
```

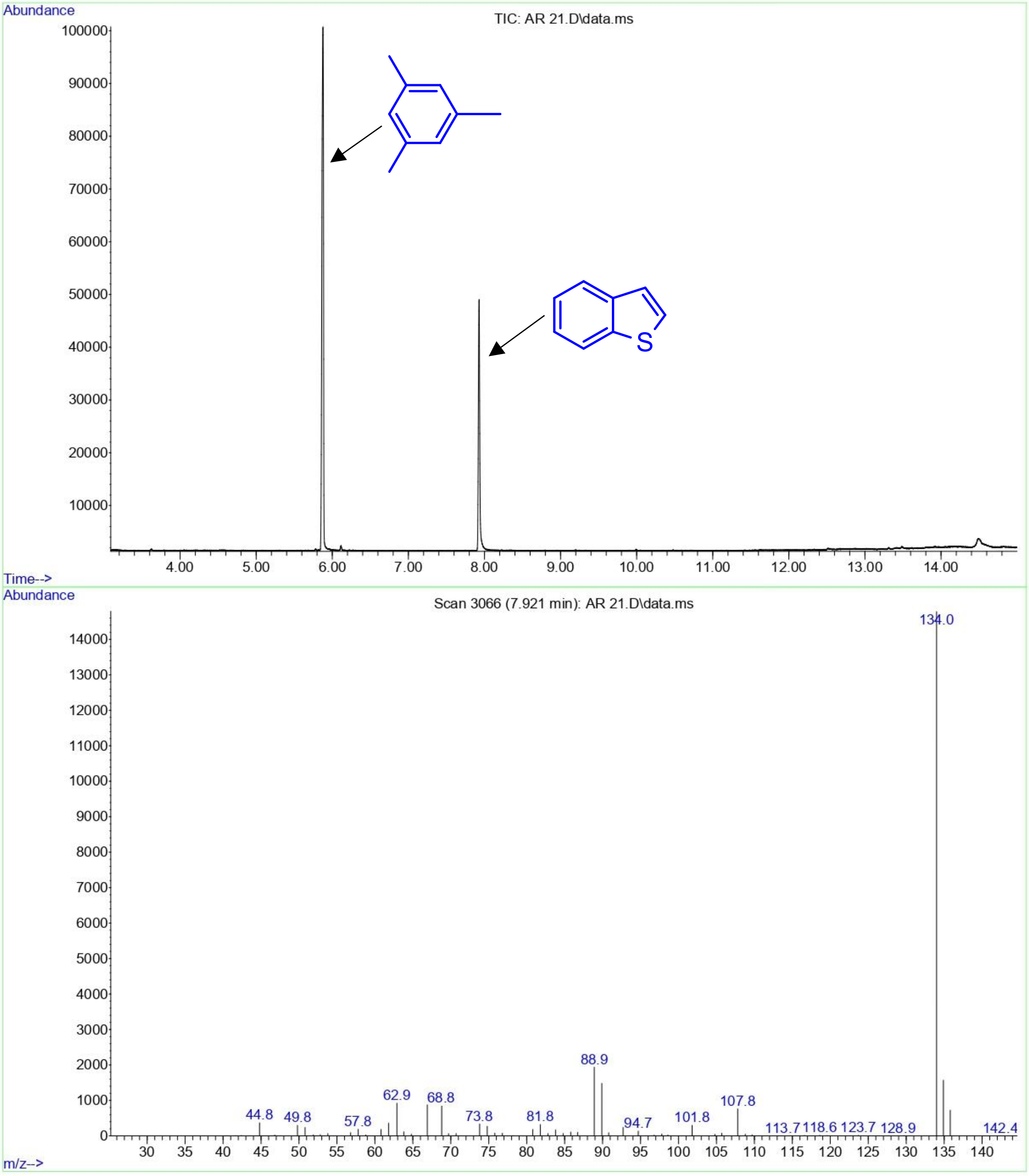

S2.13 GC–MS spectrum of the reaction described in Table 3, entry 11, with mesitylene as the internal standard.

```
File       :D:\Lab Sentral\Data\2022\Yudha Reserch Grup\2025\AR-39.D
Operator   :
Acquired   : 31 Jan 2025  16:16     using AcqMethod Umi.M
Instrument :    GCMS
Sample Name:
Misc Info  :
Vial Number: 0
```

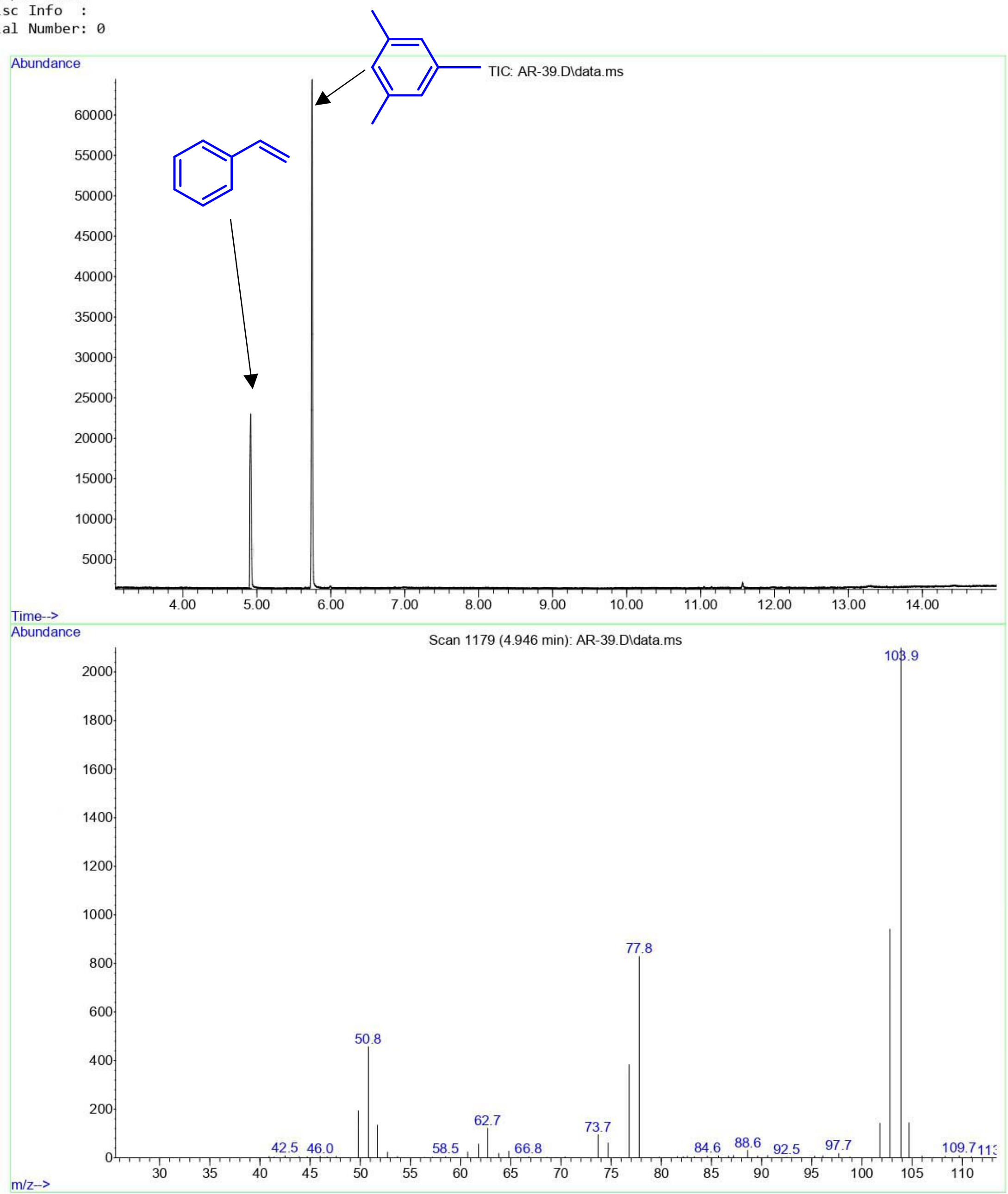

S2.14 GC–MS spectrum of the reaction described in Table 3, entry 12, with mesitylene as the internal standard.

```
File       :D:\Lab Sentral\Data\2022\Yudha Reserch Grup\2025\AR 21 ND.D
Operator   :
Acquired   : 28 Feb 2025   14:28     using AcqMethod Umi.M
Instrument :     GCMS
Sample Name:
Misc Info  :
Vial Number: 0
```

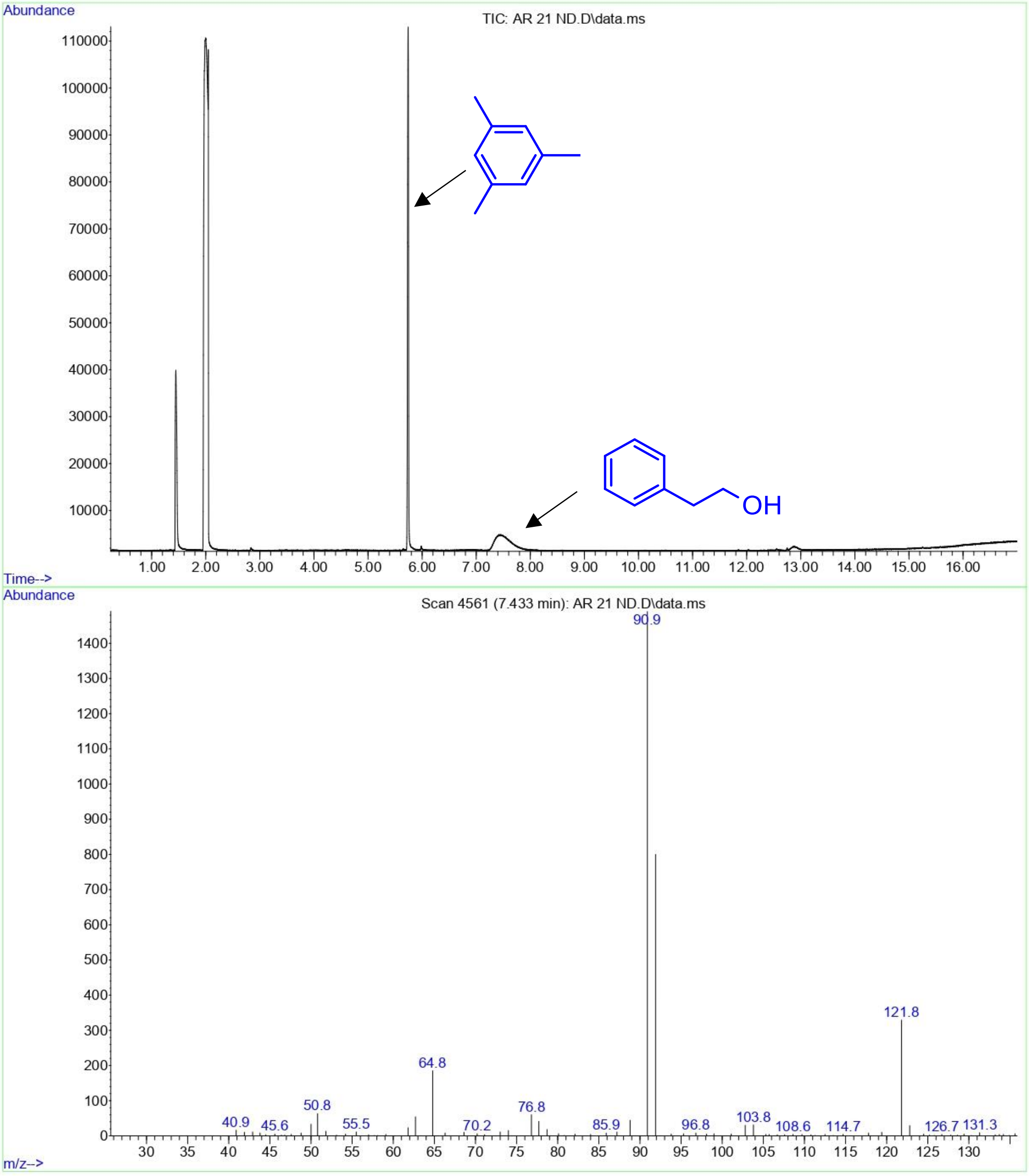

S2.15 GC–MS spectrum of the reaction described in Table 3, entry 13, with mesitylene as the internal standard.

```
File       :D:\Lab Sentral\Data\2022\Yudha Reserch Grup\2025\AR 37.D
Operator   :
Acquired   : 10 Jan 2025  14:38     using AcqMethod Umi.M
Instrument :    GCMS
Sample Name:
Misc Info  :
Vial Number: 0
```

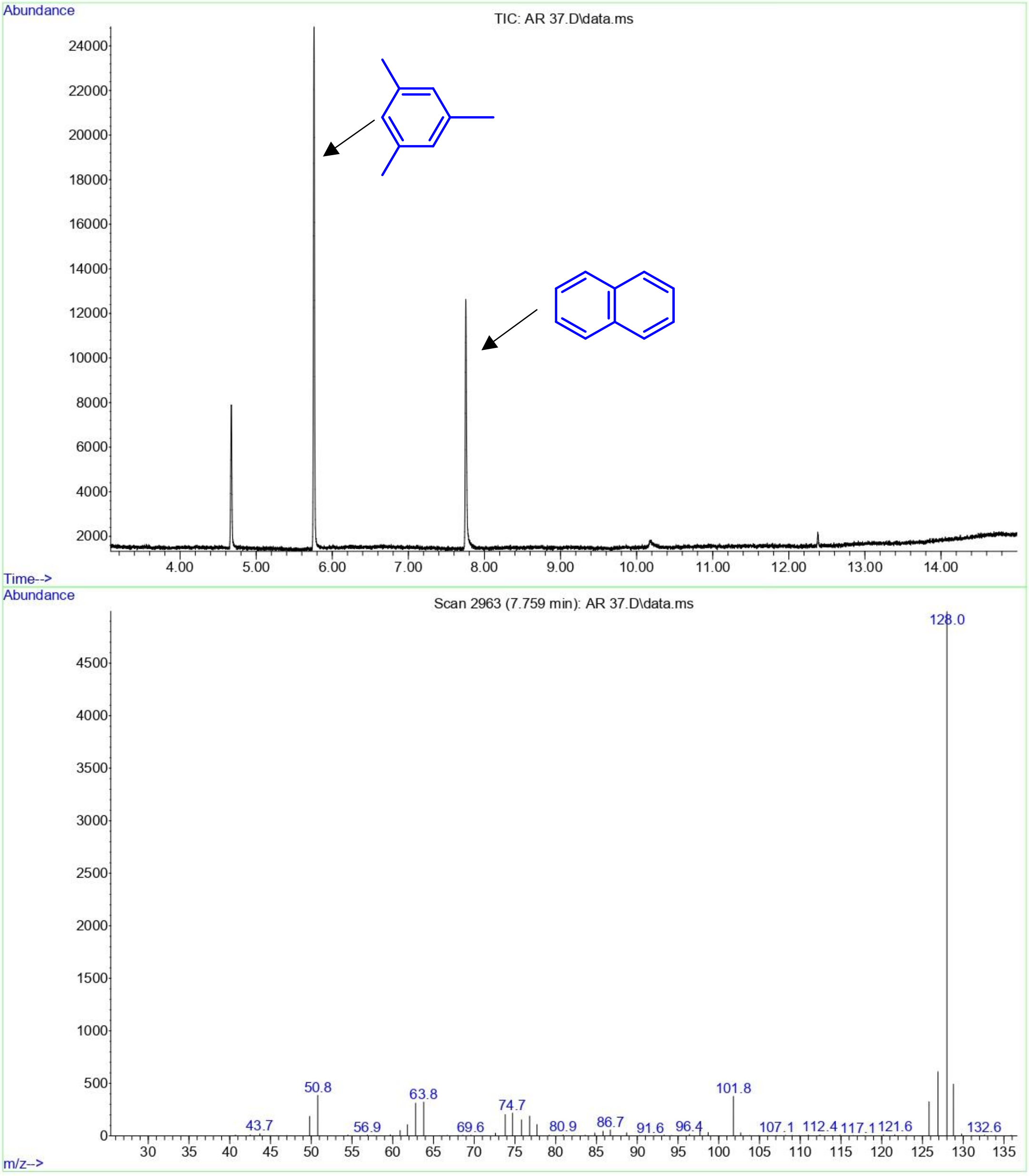

S2.16 ^{1}H NMR spectrum of **2m** (500 MHz, $CDCl_3$)

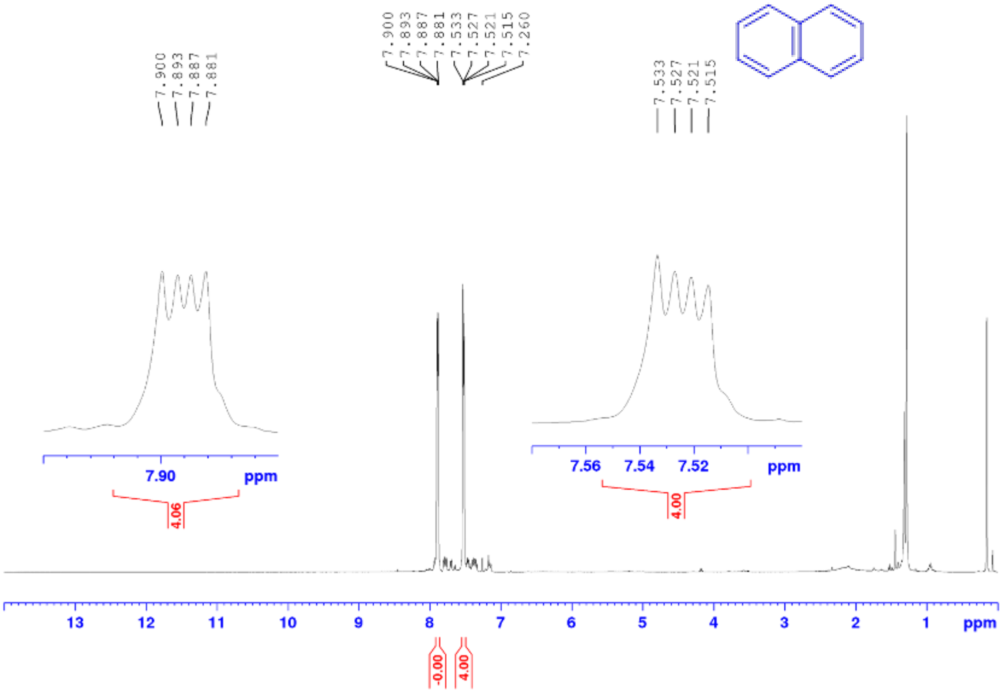

S2.17 ^{13}C{^{1}H} NMR spectrum of **2m** (125 MHz, $CDCl_3$)

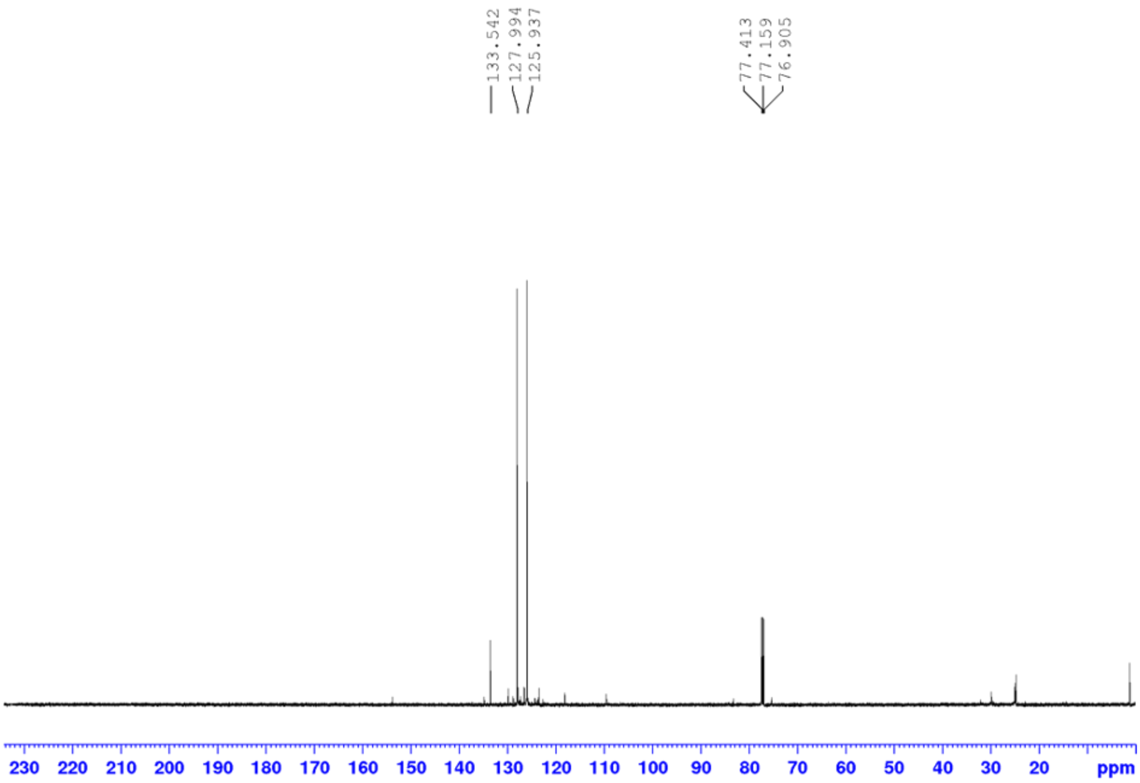

S2.18 GC–MS spectrum of the reaction described in Table 3, entry 13, with mesitylene as the internal standard.

```
File       :D:\Lab Sentral\Data\2022\Yudha Reserch Grup\2025\AR 36.D
Operator   :
Acquired   : 10 Jan 2025   14:12     using AcqMethod Umi.M
Instrument :     GCMS
Sample Name:
Misc Info  :
Vial Number: 0
```

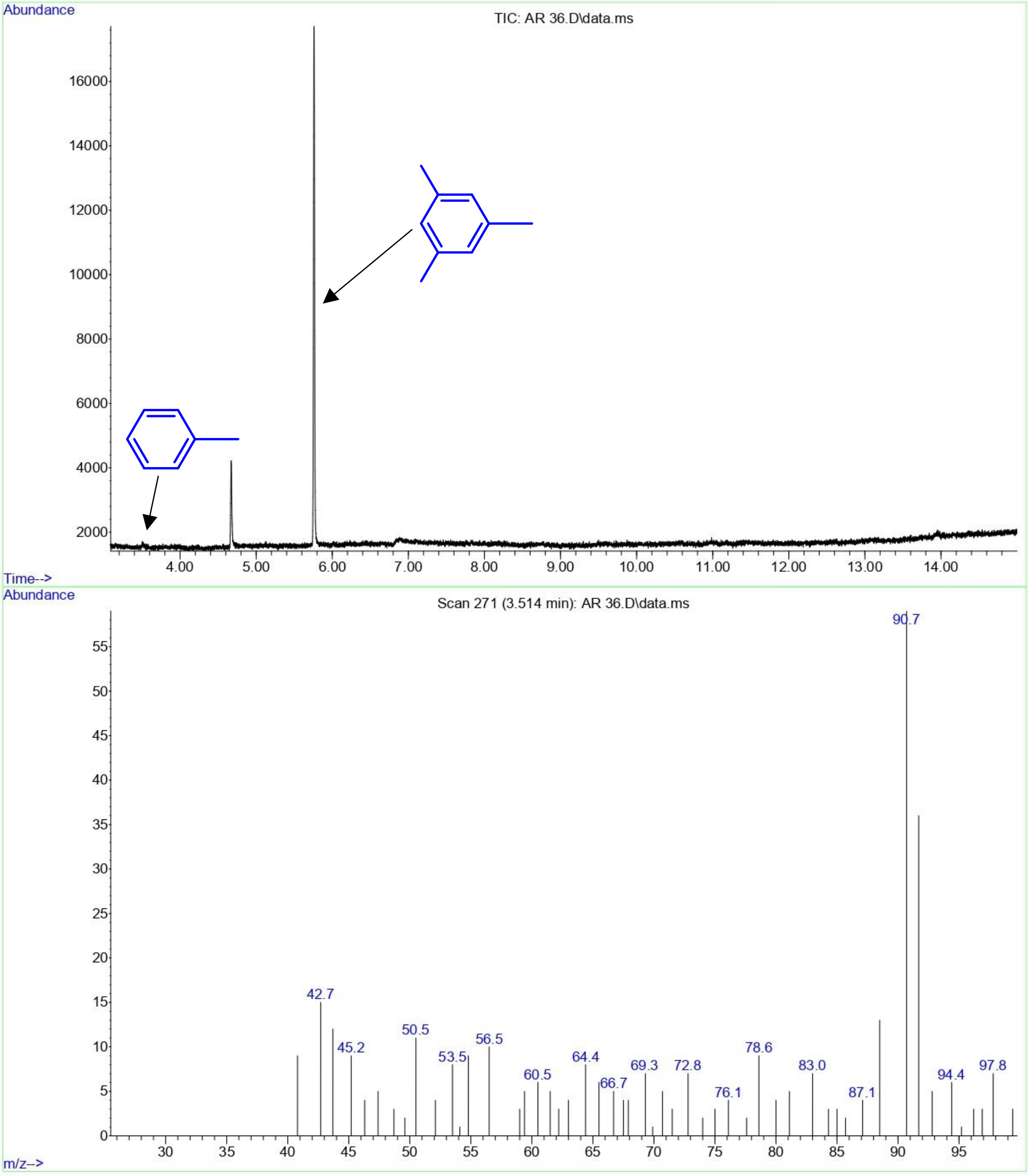

S2.19 GC-MS spectrum of stock toluene adsorption on HKUST-1 with mesitylene as an internal standard.

```
File       :D:\Lab Sentral\Data\2022\Yudha Reserch Grup\2025\AR-42.D
Operator   :
Acquired   : 24 Apr 2025  08:10     using AcqMethod Umi.M
Instrument :    GCMS
Sample Name:
Misc Info  :
Vial Number: 0
```

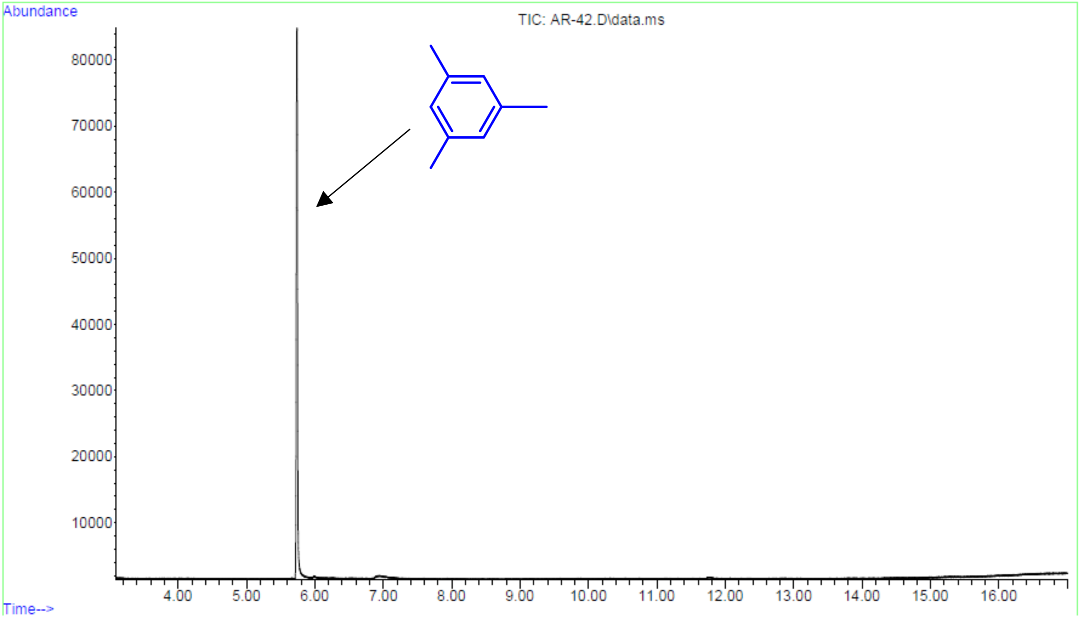

S3. XRD Data

S3.1 XRD Pattern of HKUST-1

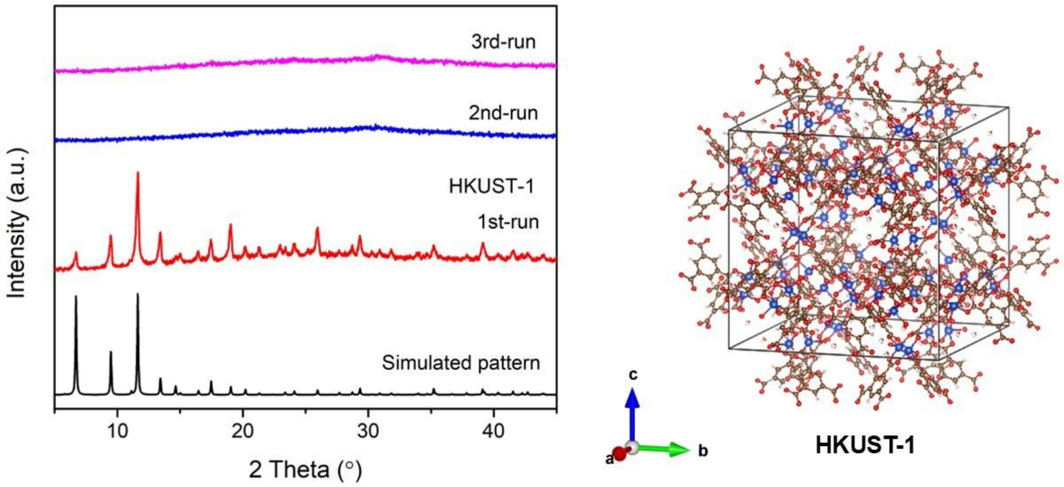

Fig. S1. Crystal structure and XRD pattern of HKUST-1 and after several uses.

S3.2 XRD Pattern of CuBDC

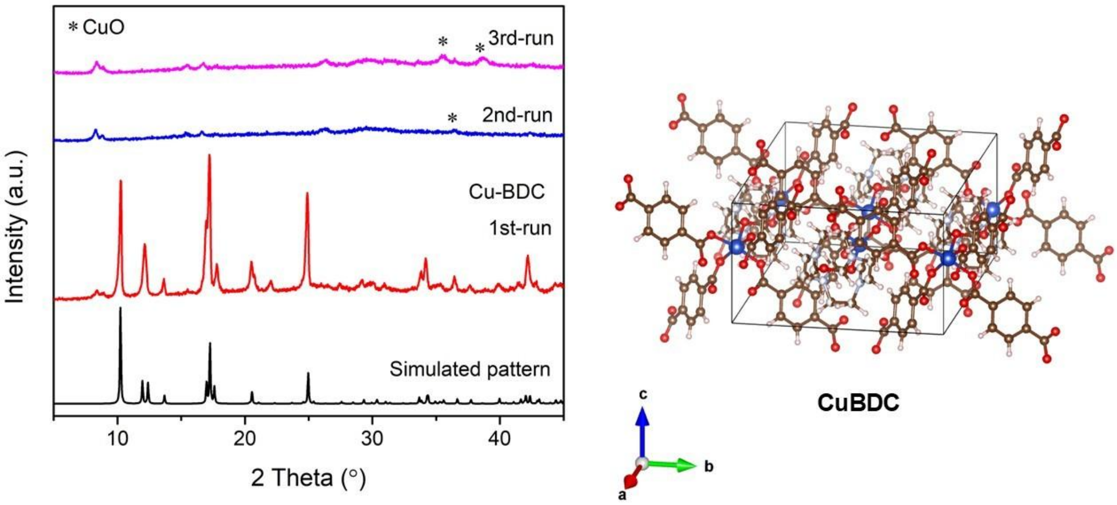

Fig. S2. Crystal structure and XRD pattern of CuBDC and after several uses.

S4. AAS Data

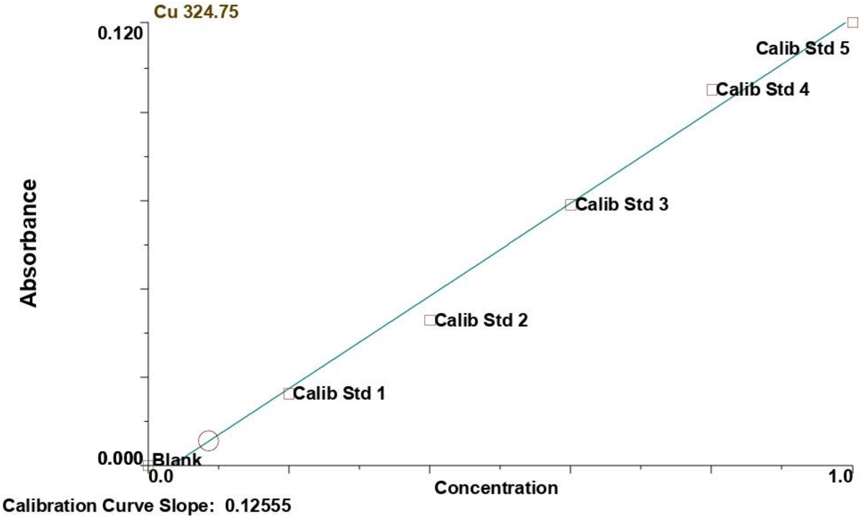

Calibration Curve Slope: 0.12555
Calibration Curve Intercept: -0.00418
Calibration Curve Correlation Coefficient: 0.995663
Calibration Curve Type: Linear, Calculated Intercept
Current Sample Concentration: 0.086 mg/L

Std #	Standard ID	Entered Conc.	Calculated Conc.	Action
Blank	Blank	0	0.033	Include
1	Calib Std 1	0.2	0.189	Include
2	Calib Std 2	0.4	0.347	Include
3	Calib Std 3	0.6	0.596	Include
4	Calib Std 4	0.8	0.845	Include
5	Calib Std 5	1.0	0.990	Include

The detected concentrations of nitric acid and ethyl acetate in the sample were 0.002 ppm and 0.003 ppm, respectively. Given a total sample concentration of 0.086 ppm, the measured copper concentration was 0.081 ppm.

Calibration data for Cu 324.75 — Equation: Linear, Calculated Intercept

ID	Mean Signal (Abs)	Entered Conc. mg/L	Calculated Conc. mg/L	Standard Deviation	%RSD
Blank	0.0000	0	0.033	0.00	7.2
Calib Std 1	0.0195	0.2	0.189	0.00	1.0
Calib Std 2	0.0394	0.4	0.347	0.00	2.2
Calib Std 3	0.0706	0.6	0.596	0.00	1.0
Calib Std 4	0.1019	0.8	0.845	0.00	0.3
Calib Std 5	0.1201	1.0	0.990	0.00	0.4

Correlation Coef.: 0.995663 Slope: 0.12555 Intercept: -0.00418

Sequence No.: 25 — Autosampler Location:
Sample ID: Blank — Date Collected: 2/24/2025 10:31:17 AM
Analyst: — Data Type: Original

Replicate Data: Blank

Repl #	SampleConc mg/L	StndConc mg/L	BlnkCorr Signal	Time	Signal Stored
1	0.026	0.026	-0.001	10:31:18	No
2	0.006	0.006	-0.003	10:31:22	No
3	0.005	0.005	-0.004	10:31:27	No
Mean:	0.012	0.012	-0.003		
SD:	0.012	0.012	0.0015		
%RSD:	99.58	99.58	58.26		

Sequence No.: 26 — Autosampler Location:
Sample ID: Blank — Date Collected: 2/24/2025 10:31:34 AM
Analyst: — Data Type: Original

Replicate Data: Blank

Repl #	SampleConc mg/L	StndConc mg/L	BlnkCorr Signal	Time	Signal Stored
1	0.003	0.003	-0.004	10:31:34	No
2	0.002	0.002	-0.004	10:31:38	No
3	0.002	0.002	-0.004	10:31:42	No
Mean:	0.002	0.002	-0.004		
SD:	0.001	0.001	0.0001		
%RSD:	25.65	25.65	1.83		

Sequence No.: 27 — Autosampler Location:
Sample ID: EA — Date Collected: 2/24/2025 10:31:56 AM
Analyst: — Data Type: Original

Replicate Data: EA

Repl #	SampleConc mg/L	StndConc mg/L	BlnkCorr Signal	Time	Signal Stored
1	0.006	0.006	-0.003	10:31:57	No
2	0.004	0.004	-0.004	10:32:02	No
3	0.005	0.005	-0.004	10:32:06	No
Mean:	0.005	0.005	-0.004		
SD:	0.001	0.001	0.0001		
%RSD:	22.22	22.22	3.74		

Sequence No.: 28 — Autosampler Location:
Sample ID: sample — Date Collected: 2/24/2025 10:32:20 AM
Analyst: — Data Type: Original

Replicate Data: sample

Repl #	SampleConc mg/L	StndConc mg/L	BlnkCorr Signal	Time	Signal Stored
1	0.087	0.087	0.007	10:32:20	No
2	0.086	0.086	0.007	10:32:25	No
3	0.086	0.086	0.007	10:32:29	No
Mean:	0.086	0.086	0.007		
SD:	0.001	0.001	0.0001		
%RSD:	1.067	1.067	1.74		

References

1. C. Liu, X. Li, Y. Wu and J. Qiu, *RSC Adv.*, 2014, **4**, 54307–54311.
2. Y.P. Budiman, M. Rashifari, S. Azid, I.Z. Ghafara, Y. Deawati, Y. Permana, U.S.F. Arrozi, W. Ciptonugroho, T. Mayanti and W.W. Lestari, *ChemistrySelect*, 2024, **9**, e202304913.
3. Z. Gai, B. Yu, X. Wang, Z. Deng and P. Xu, *Microbiology*, 2008, **154**, 3804–3812.
4. S. Abrantes, *J. High Resol. Chromatogr.*, 1993, **16**, 113–115.
5. B.M. El-Haj, A.M. Al-Amri, M.H. Hassan, R.K. Bin-Khadem and A.A. Al-Hadi, *J. Anal. Toxicol.*, 2000, **24**, 390–394.
